# The autophagy gene *Atg16l1* differentially regulates T_reg_ and T_H_2 cells to control intestinal inflammation

**DOI:** 10.7554/eLife.12444

**Published:** 2016-02-24

**Authors:** Agnieszka M Kabat, Oliver J Harrison, Thomas Riffelmacher, Amin E Moghaddam, Claire F Pearson, Adam Laing, Lucie Abeler-Dörner, Simon P Forman, Richard K Grencis, Quentin Sattentau, Anna Katharina Simon, Johanna Pott, Kevin J Maloy

**Affiliations:** 1 Sir William Dunn School of Pathology, University of Oxford, Oxford, United Kingdom; 2 Immunity at Barrier Sites Initiative, National Institute of Allergy and Infectious Diseases, National Institutes of Health, Bethesda, United States; 3 MRC Human Immunology Unit, Weatherall Institute of Molecular Medicine, University of Oxford, Oxford, United Kingdom; 4 John Radcliffe Hospital, University of Oxford, Oxford, United Kingdom; 5 Kennedy Institute of Rheumatology, University of Oxford, Oxford, United Kingdom; 6 Peter Gorer Department of Immunobiology, King's College London, London, United Kingdom; 7 Faculty of Life Sciences, The University of Manchester, Manchester, United Kingdom; Goethe University Medical School, Germany

**Keywords:** autophagy, IBD, mucosal immunology, T helper cells, Mouse

## Abstract

A polymorphism in the autophagy gene *Atg16l1* is associated with susceptibility to inflammatory bowel disease (IBD); however, it remains unclear how autophagy contributes to intestinal immune homeostasis. Here, we demonstrate that autophagy is essential for maintenance of balanced CD4^+^ T cell responses in the intestine. Selective deletion of *Atg16l1* in T cells in mice resulted in spontaneous intestinal inflammation that was characterized by aberrant type 2 responses to dietary and microbiota antigens, and by a loss of Foxp3^+^ T_reg_ cells. Specific ablation of *Atg16l1* in Foxp3^+^ T_reg_ cells in mice demonstrated that autophagy directly promotes their survival and metabolic adaptation in the intestine. Moreover, we also identify an unexpected role for autophagy in directly limiting mucosal T_H_2 cell expansion. These findings provide new insights into the reciprocal control of distinct intestinal T_H_ cell responses by autophagy, with important implications for understanding and treatment of chronic inflammatory disorders.

**DOI:** http://dx.doi.org/10.7554/eLife.12444.001

## eLife digest

The gut presents a puzzle to our immune system. Immune cells must rapidly respond to antigens produced by harmful bacteria, but food and the beneficial bacteria that inhabit the gut also produce antigens that our immune system must tolerate. Inappropriate immune responses in the gut can lead to inflammatory bowel disease, a debilitating disease with no current cure. We do not fully understand why these harmful inflammatory responses arise, but we know that genetic factors are important. Mutations in genes that affect a process known as autophagy – a pathway that breaks down and recycles unwanted material inside cells – make inflammatory bowel disease more likely to develop, but exactly how they do so remains unclear.

T helper cells are crucial controllers of intestinal immune responses and changes in their numbers and behaviour occur during inflammatory bowel disease. Kabat *et al*. explored how the autophagy pathway affects these key immune cells in mice. Blocking autophagy in T cells altered the balance of different types of T helper cells in the gut. A crucial population of regulatory T cells, which keep inflammatory responses in check, was lost. At the same time, another population of T cells expanded: the T helper 2 (T_H_2) cells that are responsible for driving allergies. As a result, the mice developed intestinal inflammation and produced antibodies against gut bacteria and food.

Overall, Kabat *et al.*’s results show that autophagy defects can alter the balance of different types of T cells in the gut, leading to inflammation in the intestine. These observations contribute to our understanding of how genetic changes may influence susceptibility to inflammatory bowel disease. They also suggest that drugs that activate autophagy could help to treat diseases associated with changes in regulatory T cells or T_H_2 cells, including inflammatory bowel disease and allergies. It will now be important to test this and to confirm whether similar changes in T cells are present in humans that have mutations in autophagy genes.

**DOI:** http://dx.doi.org/10.7554/eLife.12444.002

## Introduction

Crohn’s disease (CD) and ulcerative colitis (UC) are the two most common forms of inflammatory bowel disease (IBD), characterized by chronic inflammation of the gastrointestinal tract. IBD is a complex multifactorial disease that emerges on a background of many genetic and environmental factors (Maloy and Powrie, 2011). In recent years, tremendous efforts have been undertaken to identify the genetic factors that influence susceptibility to IBD. In particular, genome-wide association studies (GWAS) and subsequent meta-analyses have identified over 150 distinct loci that influence IBD susceptibility, many of which have revealed novel pathways in disease pathogenesis (Van Limbergen et al., 2014). Among these, a single-nucleotide polymorphism (SNP) in the essential macroautophagy (hereafter called 'autophagy') gene *ATG16L1* was associated with an increased risk of CD (Hampe et al., 2007; Rioux et al., 2007). A recent study showed that the IBD predisposing T300A mutation in the coding region of *ATG16L1* led to increased degradation of ATG16L1 protein and reduced autophagy (Murthy et al., 2014), indicating that decreased autophagy may contribute to IBD development. Polymorphisms in several other autophagy-related genes, including *IRGM, LRRK2* and *SMURF1*, are also linked to IBD susceptibility (Van Limbergen et al., 2014), suggesting that changes in the autophagy pathway alter intestinal homeostasis and predispose to chronic intestinal inflammation.

Autophagy is a highly conserved cellular process that targets cytoplasmic components for lysosomal degradation and maintains homeostasis by recycling damaged organelles and large cytoplasmic protein aggregates. Autophagy becomes particularly important during metabolic or infectious stress (Mizushima, 2007). Atg16l1 forms an essential autophagy complex with Atg5 and Atg12 that facilitates elongation of the initial isolation membrane that results in engulfment of the cargo and formation of the autophagosome. Subsequent fusion with the lysosome facilitates degradation and allows nutrient recycling (Mizushima et al., 2003). To identify the mechanisms through which autophagy may regulate intestinal tissue homeostasis, it is essential to understand the functional consequences of alterations in autophagy on both immune and tissue cells present in the gut. To date, several studies have examined the role of autophagy and Atg16l1 in intestinal epithelial cells and myeloid cells for intestinal homeostasis. In these studies, Atg16l1 was shown to play a role in Paneth cell physiology, as well as in bacterial handling and regulation of inflammatory IL-1β secretion by myeloid cells (Cadwell et al., 2008; Kuballa et al., 2008; Saitoh et al., 2008; Plantinga et al., 2011). However, the role of Atg16l1 in intestinal adaptive immune responses has not yet been addressed.

CD4^+^ T cells constitute the largest population of intestinal lymphocytes and are central mediators of host protective and tolerogenic responses in the gut (Shale et al., 2013). In particular, thymus-derived and peripherally induced Foxp3^+^ CD4^+^ regulatory T cells (tT_reg_ and pT_reg_ cells, respectively) are indispensable in promoting tolerance toward commensal and dietary antigens and for the prevention of aberrant effector T cell responses, including T_H_1, T_H_2 and T_H_17 cell responses (Izcue et al., 2009). An imbalance between effector and regulatory CD4^+^ T cells can promote chronic intestinal inflammation and accumulation of effector CD4^+^ T cells in the inflamed mucosa is a cardinal feature of IBD (Abraham and Cho, 2009; Maloy and Powrie, 2011; Shale et al., 2013). Therefore, it is important to define factors that regulate aberrant CD4^+^ T cell responses in the gastrointestinal tract.

Previous studies utilizing mice with T-cell-specific deletion of essential autophagy genes (*Atg3, Atg5, Atg7, Beclin1*) pointed to a key role of autophagy in T cell homeostasis, as these mice exhibited decreased frequencies and numbers of CD4^+^ and CD8^+^ T cells and defects in T cell proliferation in vitro (Pua et al., 2009; Stephenson et al., 2009; Jia and He, 2011; Kovacs et al., 2012). In addition, recent studies highlighted the importance of autophagy in the development of memory CD8^+^ T cells (Puleston et al., 2014; Xu et al., 2014; Schlie et al., 2015). However, the exact requirements for autophagy during different stages of T cell activation and differentiation remain poorly understood (Xu et al., 2014). Given that the gastrointestinal tract is a site of continuous immune activation by external antigens and is therefore a challenging environment for the adaptive immune system, we hypothesized that a selective defect in autophagy may affect intestinal T cell homeostasis.

We investigated the role of *Atg16l1* in intestinal CD4^+^ T cells by generating mice that selectively lack *Atg16l1* in T cells. Here, we show that T-cell-specific deletion of *Atg16l1* results in chronic intestinal inflammation accompanied by increased humoral responses toward commensal and dietary antigens. We further demonstrate that *Atg16l1*-deficiency has opposing effects on intestinal CD4^+^ T cells subsets; markedly enhancing T_H_2 responses whilst decreasing T_reg_ cell numbers. Through selective ablation of *Atg16l1* in T_reg_ cells, we established the importance of cell-intrinsic autophagy for intestinal T_reg_ cell homeostasis. Furthermore, through complementary in vivo approaches we show that autophagy controls T_H_2 responses through two distinct mechanisms; through a cell-intrinsic pathway and by promoting extrinsic regulation by T_reg_ cells.

## Results

### Selective deletion of *Atg16l1* in T cells results in spontaneous intestinal pathology

**Figure 1.**
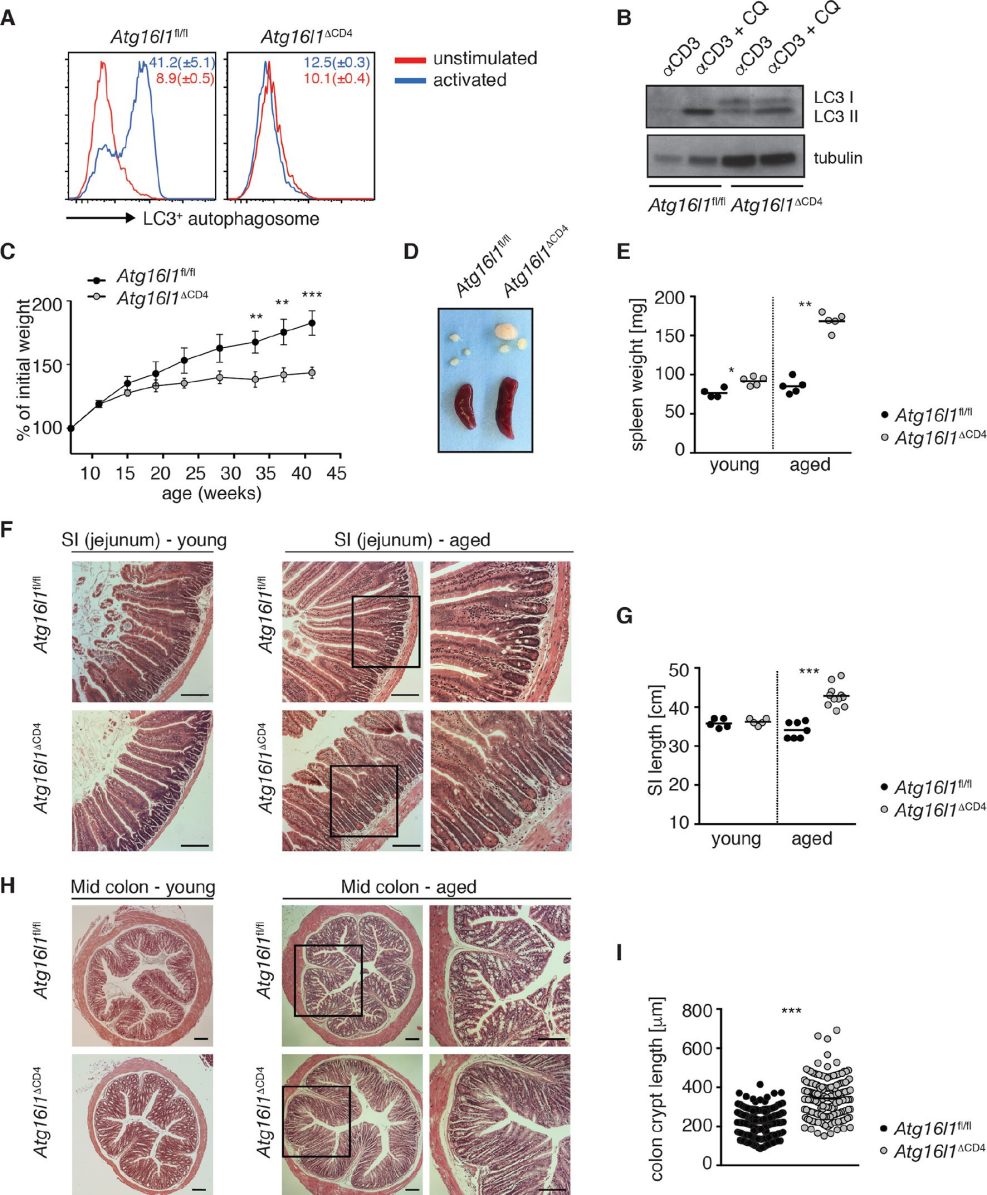
Aged *Atg16l1*^ΔCD4^ mice develop intestinal inflammation. (**A**) FACS analysis of LC3^+^ autophagosome formation in CD4^+^ T cells from cLP of *Atg16l1*^ΔCD4^ and *Atg16l1*^fl/fl^ mice after overnight activation with or without α-CD3 (5 μg/ml) and α-CD28 (1 μg/ml). (**B**) Western blot analysis of LC3 lipidation in naïve splenic CD4^+^ T cells isolated from *Atg16l1*^ΔCD4^ mice and *Atg16l1*^fl/fl^ mice after 3hr activation with α-CD3 (5 μg/ml) and α-CD28 (1 μg/ml) with or without chloroquine (CQ, inhibitor of lysosomal degradation, 50 μM). (**C**) Weight curves of *Atg16l1*^ΔCD4^ and *Atg16l1*^fl/fl^ littermates. (**D**) Representative images of spleens and mesenteric lymph nodes (mLN) from aged *Atg16l1*^ΔCD4^ and *Atg16l1*^fl/fl^ littermates and (**E**) spleen weights of young and aged *Atg16l1*^ΔCD4^ and *Atg16l1*^fl/fl^ littermates. (**F,H**) Representative photomicrographs of haemotoxilin and eosin (H&E) stained sections of (**F**) jejunum and (**H**) mid-colon from young and aged *Atg16l1*^ΔCD4^ and *Atg16l1*^fl/fl^ littermates, scale bar 150 μm. (**G,I**) Quantification of (**G**) SI lengths and (**I**) mid-colon crypt lengths in aged *Atg16l1*^ΔCD4^ and *Atg16l1*^fl/fl^ littermates. Data are representative of at least three independent experiments (**A-E, F, H**) or combined from two (**G**) or three (**I**) independent experiments, with at least 3 mice per group. Data shown as mean ± s.e.m (**A,C**). Each dot represents an individual mouse and horizontal bars denote means (**E,G**). In (**I**) each dot represents an individual crypt measurement and horizontal bars denote means. Statistical significance was determined using two-way analysis of variance (ANOVA) with Bonferroni’s correction for multiple comparisons (**C**) or the Mann–Whitney test (**E,G,I**), **p<0.01; ***p<0.001. SI LP– small intestine lamina propria, cLP – colonic lamina propria. Young mice: 8–12 weeks old, aged mice >5 months old. **DOI:** http://dx.doi.org/10.7554/eLife.12444.003

To investigate the role of autophagy in intestinal T cell homoeostasis, mice carrying *lox*P-flanked alleles of the essential autophagy gene *Atg16l1 (Atg16l1*^fl/fl^) (Hwang et al., 2012) were crossed with *CD4-Cre* mice, generating *Atg16l1*^fl/fl^::*CD4-Cre* mice (hereafter denoted as *Atg16l1*^ΔCD4^) in which *Atg16l1* is selectively ablated in T cells from the double-positive stage of thymic development. To verify functional deletion of *Atg16l1* autophagy levels were analyzed by autophagosome formation and LC3 lipidation. CD4^+^ T cells isolated from control *Atg16l1*^fl/fl^ mice exhibited increased LC3^+^ autophagosome formation after activation, as measured by intracellular LC3 accumulation in the presence of a lysosomal inhibitor (Figure 1A). In contrast, there was no increase in intracellular LC3 accumulation in CD4^+^ T cells from *Atg16l1*^ΔCD4^ mice (Figure 1A). To verify this finding using another method, we assessed LC3 lipidation by Western blot analysis (Klionsky et al., 2012). Activated control *Atg16l1*^fl/fl^ CD4^+^ T cells exhibited increased lipidated LC3 II levels in the presence of chloroquine, indicative of autophagy-mediated turnover of LC3 II after T cell activation (Figure 1B). However, LC3 II levels in CD4^+^ T cells from *Atg16l1*^ΔCD4^ mice were barely affected by activation (Figure 1B), confirming a block in autophagy.

Young *Atg16l1*^ΔCD4^ mice appeared normal, initially gained weight in a manner comparable to *Atg16l1*^fl/fl^ littermates and exhibited normal intestinal morphology (Figure 1C,F-H). However, from around 5 months of age, *Atg16l1*^ΔCD4^ mice stopped gaining weight (Figure 1C), developed splenomegaly and lymphadenopathy (Figure 1D,E) and chronic intestinal pathology that progressed with age (Figure 1F–I). *Atg16l1*^ΔCD4^ mice exhibited significant inflammation of both the small intestine (SI) and colon, characterized by increased SI length, marked lengthening of crypts, shortening of villi and epithelial hyperplasia (Figure 1F–I). Thus, T-cell-specific *Atg16l1* deletion resulted in spontaneous intestinal inflammation and systemic immune activation.

### *Atg16l1* deficiency has opposing effects on intestinal T_reg_ and T_H_2 cells

**Figure 2.**
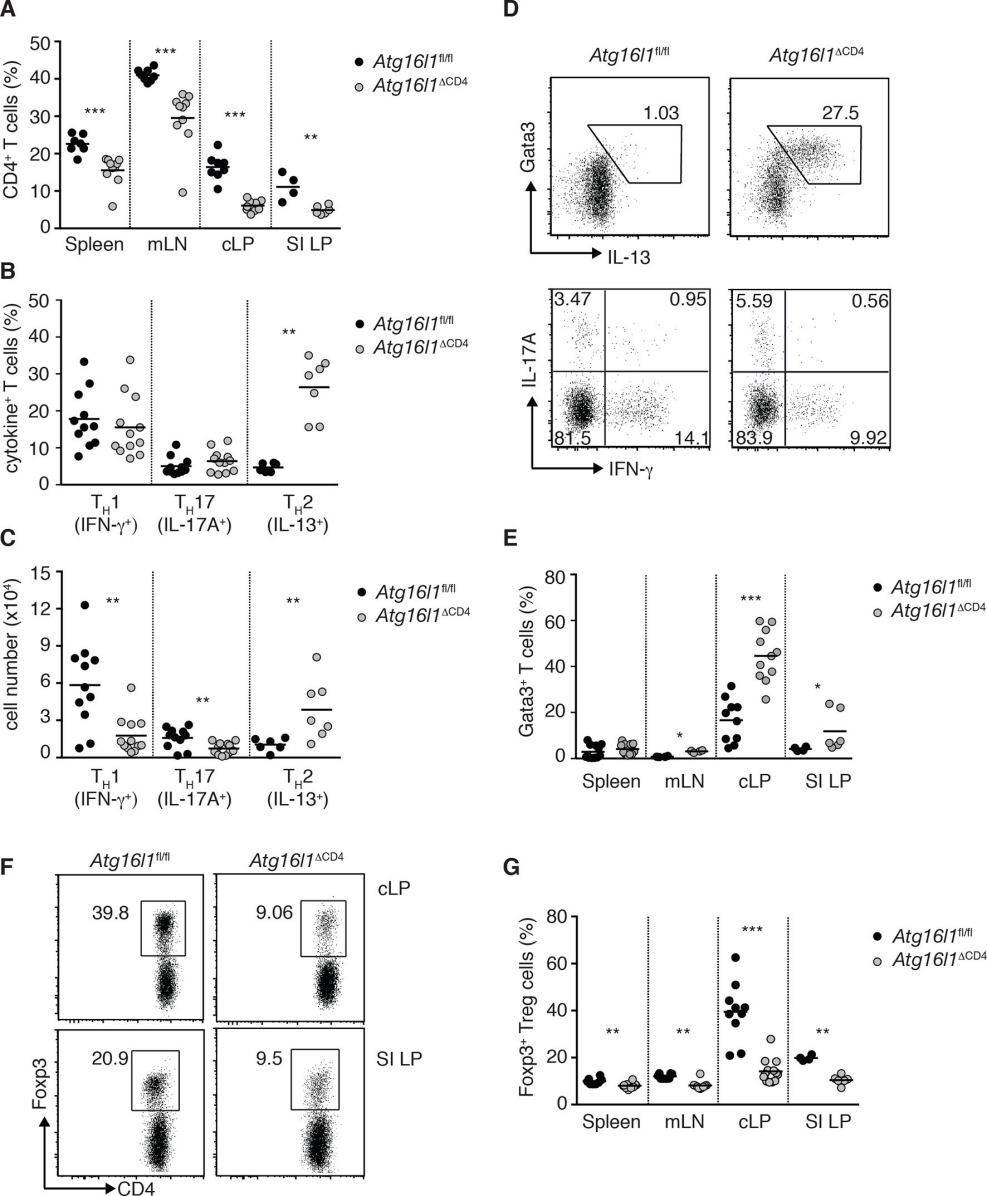
*Atg16l1*^ΔCD4^ mice exhibit reciprocal dysregulation of intestinal T_H_2 and T_reg_ cells before the onset of intestinal inflammation. (**A**) Frequencies of CD4^+^ T cells as a proportion of live cells in young *Atg16l1*^ΔCD4^ and *Atg16l1*^fl/fl^ littermates. (**B**) Frequencies and (**C**) total numbers of IFN-γ^+^ T_H_1, IL-17A^+^ T_H_17 and IL-13^+^ T_H_2 cells isolated from cLP of young *Atg16l1*^ΔCD4^ and *Atg16l1*^fl/fl^ littermates (gated on CD4^+^ T cells). (**D**) Representative FACS plots of Gata3 and IL-13 (top) or IFN-γ and IL-17A (bottom) expression by cLP CD4^+^ T cells isolated from young *Atg16l1*^ΔCD4^ and *Atg16l1*^fl/fl^ littermates (gated on CD4^+^ TCRβ^+^ Foxp3^-^ live cells). (**E**) Frequencies of Gata3^+^ CD4^+^ T cells in young *Atg16l1*^ΔCD4^ and *Atg16l1*^fl/fl^ littermates (gated on CD4^+^ TCRβ^+^ Foxp3^-^ cells). (**F**) Representative FACS plots and (**G**) frequencies of Foxp3^+^ T_reg_ cells in young *Atg16l1*^ΔCD4^ and *Atg16l1*^fl/fl^ littermates (gated on CD4^+^ TCRβ^+^ cells). Data are combined from three or more independent experiments with at least two mice per group (**A,B, D, E, G**) or are representative of four independent experiments with at least four mice per group (**D, F**). Each dot represents an individual mouse and horizontal bars denote means. Numbers indicate percentage of cells in gates or quadrants. Statistical significance was determined using the Mann–Whitney test, *p<0.05; **p<0.01; ***p<0.001. SI LP– small intestine lamina propria, cLP – colonic lamina propria. Young mice: 8–12 weeks old. **DOI:** http://dx.doi.org/10.7554/eLife.12444.004

To characterize the effects of *Atg16l1* on intestinal and systemic T cell homeostasis independently from any confounding effects of ongoing tissue inflammation, we analyzed young (8–12 weeks old) *Atg16l1*^ΔCD4^ mice before the onset of inflammatory pathology or systemic symptoms. Whilst thymic T cell production was unperturbed in *Atg16l1*^ΔCD4^ mice (Figure 2—figure supplement 1A,B), frequencies of CD4^+^ and CD8^+^ T cells in peripheral lymphoid organs were significantly decreased compared to *Atg16l1*^fl/fl^ littermates (Figure 2A and Figure 2—figure supplement 1C). Furthermore, we observed significant decreases in intestinal T cell frequencies and numbers in the cLP and SI LP of *Atg16l1*^ΔCD4^ mice (Figure 2A and Figure 2—figure supplement 1D). As CD4^+^ T cells are the main drivers and regulators of chronic intestinal inflammation (Shale et al., 2013), we focused subsequent analyses on CD4^+^ T cells.

**Figure 2—figure supplement 1.**
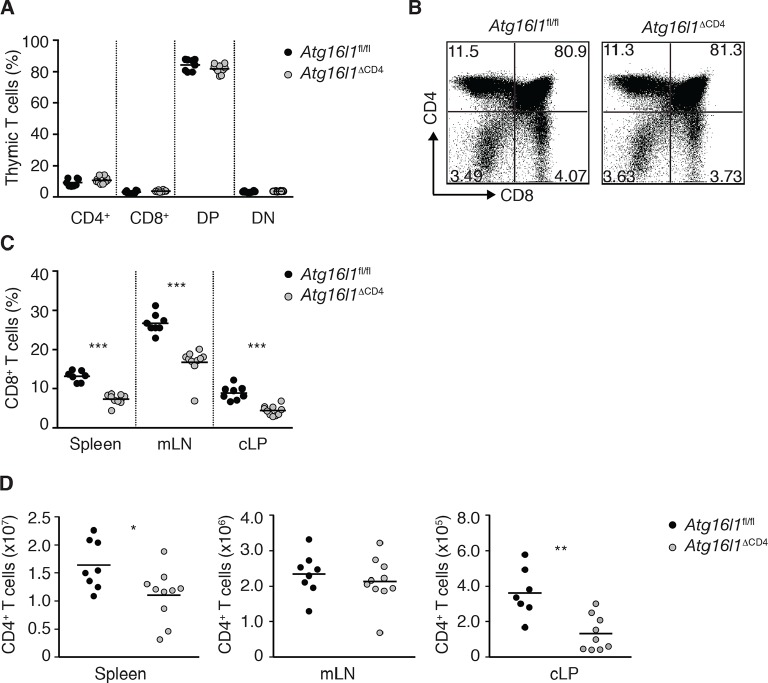
Characterization of immune cell compartments in young *Atg16l1*^ΔCD4^mice. (**A**) Frequencies and (**B**) representative FACS plots of single positive CD4^+^, single positive CD8^+^, double positive (DP) CD4^+^ CD8^+^ and double negative (DN) CD4^-^ CD8^-^ thymocytes in young *Atg16l1*^ΔCD4^ and *Atg16l1*^fl/fl^ littermates. (**C**) Frequencies of CD8^+^ T cells in young *Atg16l1*^ΔCD4^ and *Atg16l1*^fl/fl^ littermates. (**D**) Total numbers of CD4^+^ T cells in spleen, mLN, and cLP of young *Atg16l1*^ΔCD4^ and *Atg16l1*^fl/fl^ littermates. Data are combined from (**A,C,D**) or representative of (**B**) two or three independent experiments with at least 4 mice per group. Each dot represents an individual mouse and horizontal bars denote means. Numbers indicate percentage of cells in quadrants. Statistical significance was determined using the Mann–Whitney test, *p<0.05; **p<0.01; ***p<0.001. cLP – colonic lamina propria, mLN - mesenteric lymph nodes. Young mice: 8–12 weeks old. **DOI:** http://dx.doi.org/10.7554/eLife.12444.005

**Figure 2—figure supplement 2.**
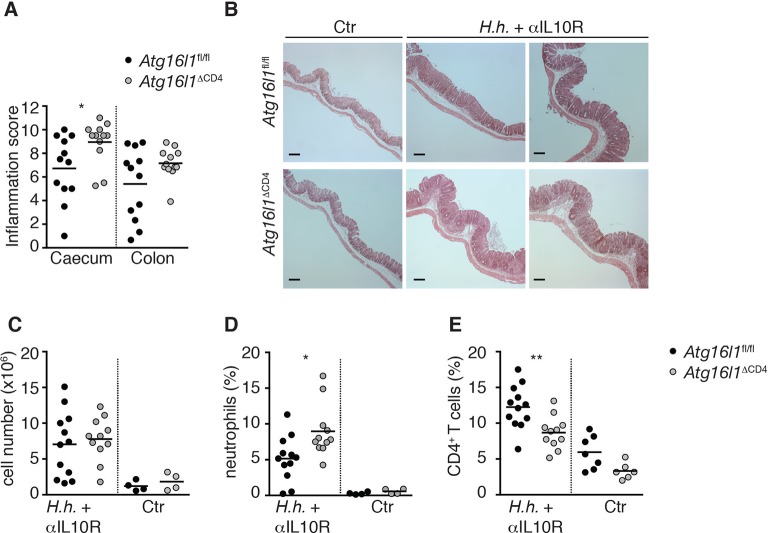
*Atg16l1*^ΔCD4^ mice have increased susceptibility to T-cell-mediated experimental IBD. Cohorts of young *Atg16l1*^ΔCD4^ and *Atg16l1*^fl/fl^ littermates were infected with *Helicobacter hepaticus* by oral gavage (three feeds of 1x10^8^ CFU) and treated with anti-IL-10R mAb (1mg/mouse *i.p*. given weekly; *H.h* + αIL10R) or left untreated (Ctr). Two weeks post-infection mice were sacrificed for analyses. (**A**) Caecum and colon histopathology scores in *H.h* + αIL10R-treated *Atg16l1*^ΔCD4^ and *Atg16l1*^fl/fl^ littermates. (**B**) Representative photomicrographs of H&E stained caecum of untreated Ctr (left panels) or *H.h* + αIL10R-treated (middle and right panels) *Atg16l1*^ΔCD4^ and *Atg16l1*^fl/fl^ littermates, scale bar 150 μm. (**C**) Total lamina propria leukocyte numbers and (**D,E**) frequencies of (**D**) neutrophils (Gr1^hi^ CD11b^+^) and (**E**) CD4^+^ T cells in cLP isolated from untreated Ctr or *H.h* + αIL10R-treated *Atg16l1*^ΔCD4^ and *Atg16l1^fl/fl ^*littermates. Data are combined from two or three independent experiments with at least two mice per group. Each dot represents an individual mouse and horizontal bars denote means. Statistical significance was determined using the Mann–Whitney test, *p<0.05; **p<0.01. **DOI:** http://dx.doi.org/10.7554/eLife.12444.006

**Figure 2—figure supplement 3.**
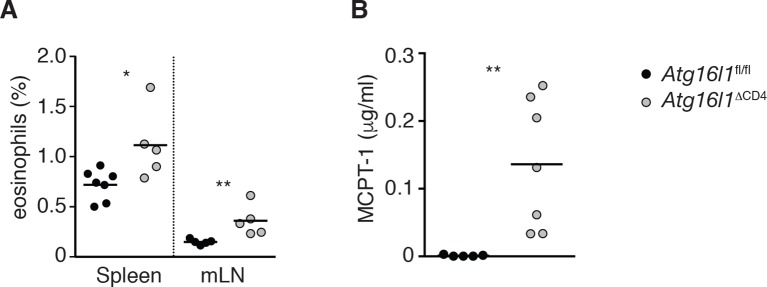
Elevated type 2 innate responses in *Atg16l1*^ΔCD4^ mice. (**A**) Frequencies of eosinophils (Ly6C^low^ Ly6G^low^ CD11b^+^ F4/80^-^) in spleen and mLN of young *Atg16l1*^ΔCD4^ and *Atg16l1*^fl/fl^ littermates. (**B**) Serum MCPT-1 levels in young *Atg16l1*^ΔCD4^ and *Atg16l1*^fl/fl^ littermates were measured by ELISA. Data are combined from (A) or representative of (B) two or three independent experiments with at least four mice per group. Each dot represents an individual mouse and horizontal bars denote means. Statistical significance was determined using the Mann–Whitney test, *p<0.05; **p<0.01. mLN - mesenteric lymph nodes. Young mice: 8–12 weeks old. **DOI:** http://dx.doi.org/10.7554/eLife.12444.007

**Figure 2—figure supplement 4.**
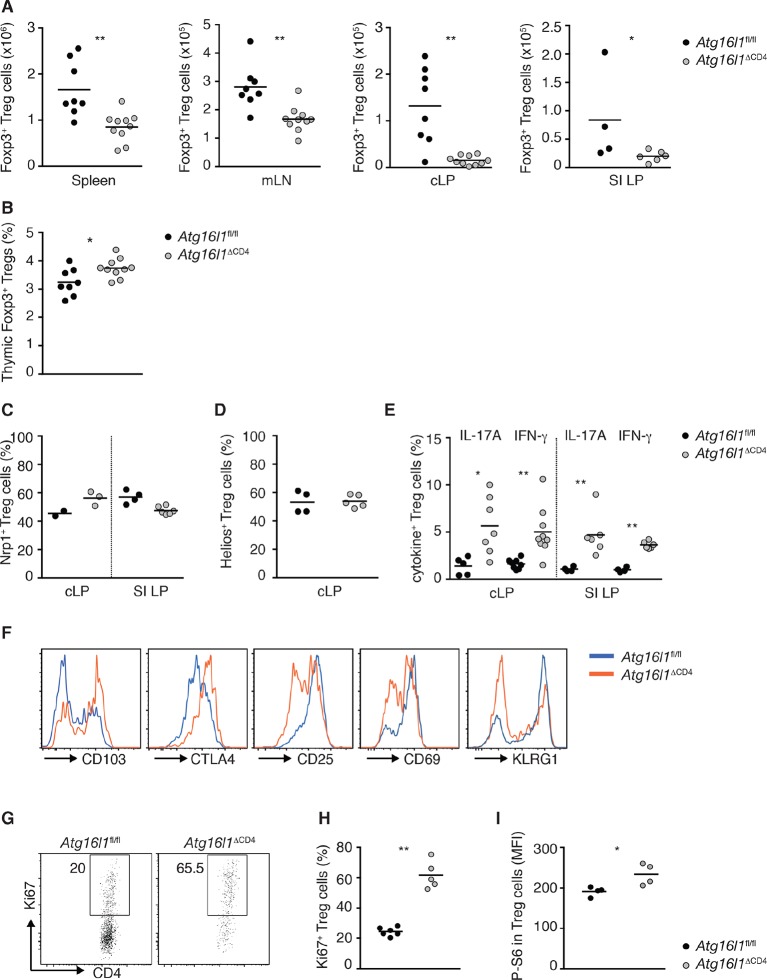
Characterization of *Atg16l1*-deficient T_reg_ cells. (**A**) Foxp3^+^ T_reg_ cell numbers in spleen, mLN, cLP and SI LP of young *Atg16l1*^ΔCD4^ and *Atg16l1*^fl/fl^ littermates. (**B**) Frequencies of Foxp3^+^ T_reg_ in the thymus of young *Atg16l1*^ΔCD4^ and *Atg16l1*^fl/fl^ littermates (gated on single positive CD4^+^ TCRβ^+^ cells). (**C**) Frequencies of Neuropilin-1^+^ (Nrp1^+^) Foxp3^+^ T_reg_ cells in the cLP and SI LP of young *Atg16l1*^ΔCD4^ and *Atg16l1*^fl/fl^ littermates (gated on CD4^+^ TCRβ^+^ Foxp3^+^ T cells). (**D**) Frequencies of Helios^+^ Foxp3^+^ T_reg_ cells in the cLP of young *Atg16l1*^ΔCD4^ and *Atg16l1*^fl/fl^ littermates (gated on CD4^+^ TCRβ^+^ Foxp3^+^ T cells). (**E**) Frequencies of IL-17A^+^ or IFN-γ^+^ of Foxp3^+^ T_reg_ cells in the cLP and SI LP of young *Atg16l1*^ΔCD4^ and *Atg16l1*^fl/fl^ littermates (gated on Foxp3^+^ CD4^+^ TCRβ^+^ T cells). (**F**) Expression of CD103, CTLA4, CD25, CD69 and KLRG1 by cLP Foxp3^+^ T_reg_ cells from young *Atg16l1*^ΔCD4^ and *Atg16l1*^fl/fl^ littermates (gated on Foxp3^+^ CD4^+^ TCRβ^+^ T cells). (**G**) Representative FACS plots and (**H**) frequencies of Ki67^+^ Foxp3^+^ T_reg_ cells in cLP of young *Atg16l1*^ΔCD4^ and *Atg16l1*^fl/fl^ littermates (gated on Foxp3^+^ CD4^+^ TCRβ^+^ T cells). (**I**) Mean fluorescence intensity (MFI) of phospo-S6 (P-S6) in Foxp3^+^ T_reg_ cells in cLP of young *Atg16l1*^ΔCD4^ and *Atg16l1*^fl/fl^ littermates. Data are combined from two independent experiments with at least four mice per group (**A,B,E**), are representative from two independent experiments with at least four mice per group (**C,F-H**), or are from one experiment (**D,I**). Each dot represents an individual mouse and horizontal bars denote means. Numbers indicate percentage of cells in gates. Statistical significance was determined using the Mann–Whitney test, *p<0.05; **p<0.01. mLN - mesenteric lymph nodes, SI LP– small intestine lamina propria, cLP – colonic lamina propria. Young mice: 8–12 weeks old. **DOI:** http://dx.doi.org/10.7554/eLife.12444.008

Despite reduced numbers of T cells, *Atg16l1*^ΔCD4^ mice developed exacerbated disease in a CD4^+^ T cell-mediated model of IBD, indicating that *Atg16l1*-deficient CD4^+^ T cells are capable of driving intestinal inflammation (Figure 2—figure supplement 2). Analysis of the effector CD4^+^ T cell compartment in *Atg16l1*^ΔCD4^ mice revealed that frequencies of colonic T_H_1 (IFN-γ^+^) and T_H_17 (IL-17A^+^) populations were comparable in young *Atg16l1*^ΔCD4^ mice and *Atg16l1*^fl/fl^ littermates (Figure 2B,D), although, due to decreased colonic CD4^+^ T cell numbers, total T_H_1 and T_H_17 numbers were significantly decreased (Figure 2C). Conversely, both frequencies and total numbers of T_H_2 (IL-13^+^) cells were significantly increased in cLP of young *Atg16l1*^ΔCD4^ mice (Figure 2B–D). These IL-13-producing cells were *bona fide* T_H_2 cells, as they co-expressed the lineage-specifying transcription factor Gata3 (Figure 2D,E). Interestingly, T_H_2 cell accumulation was primarily observed in the intestinal mucosa of *Atg16l1*^ΔCD4^ mice, as T_H_2 cell frequencies were only marginally increased in the mLN and remained unchanged in the spleen (Figure 2E). However, the functional consequences of T_H_2 expansion extended beyond the intestine, as *Atg16l1*^ΔCD4^ mice had increased frequencies of eosinophils in both the spleen and mLN and elevated serum levels of mast cell protease 1 (MCPT-1), a marker of intestinal mast cell activation (Figure 2—figure supplement 3A,B).

As Foxp3^+^ T_reg_ cells play a non-redundant role in control of effector T cells and the development of intestinal inflammation (Izcue et al., 2009), we hypothesized that alterations in T_regs_ might underlie the spontaneous intestinal pathology that developed in aged *Atg16l1*^ΔCD4^ mice. Indeed, we found that the frequencies of intestinal Foxp3^+^ T_reg_ cells in young *Atg16l1*^ΔCD4^ mice were severely reduced, both in the SI and cLP (Figure 2F,G). Taking into account the decreased frequencies of CD4^+^ T cells in *Atg16l1*^ΔCD4^ mice (Figure 2A), this equated to a reduction in T_reg_ cell numbers by around 10-fold in the colonic LP and 4-fold in SI LP (Figure 2—figure supplement 4A). In contrast, thymic development of Foxp3^+^ T_reg_ cells was not diminished in young *Atg16l1*^ΔCD4^ mice (Figure 2—figure supplement 4B), and we observed only minor, though significant, reductions in the frequencies and absolute numbers of Foxp3^+^ T_reg_ cells in the spleen and mLN of *Atg16l1*^ΔCD4^ mice compared with *Atg16l1*^fl/fl^ littermates (Figure 2G and Figure 2—figure supplement 4A). Thus, *Atg16l1*-deficiency profoundly affected the maintenance of Foxp3^+^ T_reg_ cells in the periphery, particularly within the intestinal mucosa. Expression of neuropilin-1 (Nrp1) and Helios, putative markers proposed to distinguish pT_reg_ and tT_reg_ cells, were found at comparable levels on intestinal Foxp3^+^ T_reg_ cells from *Atg16l1*^fl/fl^ and *Atg16l1*^ΔCD4^ mice, suggesting that the local environment, rather than site of T_reg_ induction, primarily dictates the requirement for autophagy in T_reg_ cells (Figure 2—figure supplement 4C,D). Assessment of how *Atg16l1*-deficiency affected intestinal Foxp3^+^ T_reg_ cell phenotype showed that impaired autophagy significantly increased expression of effector T_H_ cytokines in T_reg_ cells from cLP and SI LP (Figure 2—figure supplement 4E). We also found that cLP T_reg_ cells from young *Atg16l1*^ΔCD4^ mice showed higher expression of CD103 and CTLA-4, but showed decreased expression of the activation markers CD25, CD69, and the terminal differentiation marker KLRG-1 (Cheng et al., 2012) (Figure 2—figure supplement 4F). In addition, intestinal T_reg_ cells from young *Atg16l1*^ΔCD4^ mice had significantly increased expression of Ki67 and higher levels of phosphorylated S6, suggesting that the majority were in cell cycle (Figure 2—figure supplement 4G–I). Taken together, these results identify a crucial role for autophagy in the maintenance and functional regulation of intestinal T_reg_ cells.

Overall, these results demonstrate that selective ablation of *Atg16l1* in T cells led to a decrease in Foxp3^+^ T_reg_ cells and selective expansion of T_H_2 cells that preceded the onset of overt pathology. In addition, these perturbations in T_H_ cell subsets were largely limited to the mucosal environment.

### *Atg16l1*^ΔCD4^ mice exhibit elevated type 2 humoral responses to environmental antigens

**Figure 3.**
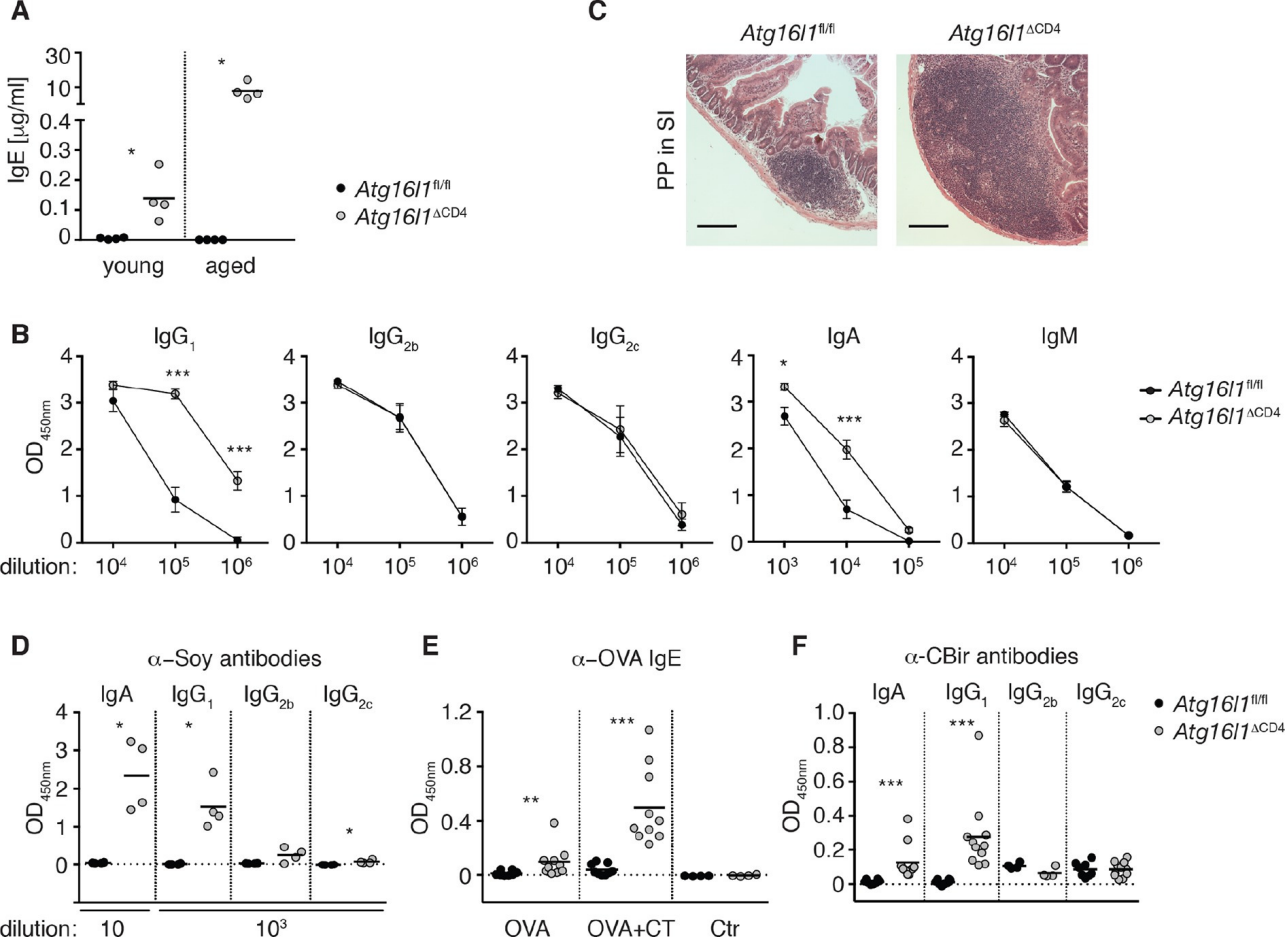
*Atg16l1*^ΔCD4^ mice develop elevated T_H_2-associated antibodies against intestinal luminal antigens. (**A**) Serum IgE concentrations in cohorts of young and aged *Atg16l1*^ΔCD4^ and *Atg16l1*^fl/fl^ littermates were measured by ELISA. (**B**) Serum antibody IgG_1_, IgG_2b_, IgG_2c_, IgA and IgM isotype levels in aged *Atg16l1*^ΔCD4^ and *Atg16l1*^fl/fl^ littermates were measured by ELISA. (**C**) Representative photomicrographs of H&E stained sections of Peyer’s patch (PP) in the SI (jejunum) of aged *Atg16l1*^ΔCD4^ and *Atg16l1*^fl/fl^ littermates, scale bar 150 μm. (**D**) Serum levels of Soy-specific IgA, IgG_1_, IgG_2b_, IgG_2c_ antibodies in aged *Atg16l1*^ΔCD4^ and *Atg16l1*^fl/fl^ littermates were measured by ELISA. (**E**) Young *Atg16l1*^ΔCD4^ and *Atg16l1*^fl/fl^ littermates were fed with ovalbumin (OVA) alone or with cholera toxin (CT) as described in methods and levels of OVA-specific serum IgE were measured 8 weeks after first challenge by ELISA. (**F**) Levels of CBir1-specific IgA, IgG_1,_ IgG_2b_ and IgG_2c_ antibodies in serum of aged *Atg16l1*^ΔCD4^ and *Atg16l1*^fl/fl^ littermates were measured by ELISA, serum was diluted 50x. Data are representative from at least two independent experiments with at least three mice per group (**A-D**) or combined from two (**E**) or three (**F**) independent experiments with at least three mice per group. Each dot represents an individual mouse and horizontal bars denote means (**A,D,E,F**). Serum isotype levels are shown as mean ± s.e.m (**B**). Statistical significance was determined using the Mann–Whitney test (**A,D-F**) or two-way analysis of variance (ANOVA) with Bonferroni’s correction for multiple comparisons (**B**), *p<0.05; **p<0.01; ***p<0.001. SI – small intestine. Young mice: 8–12 weeks old, aged mice > 5 months old. **DOI:** http://dx.doi.org/10.7554/eLife.12444.009

We next assessed whether dysregulation in the intestinal T_reg_ and T_H_2 compartment in *Atg16l1*^ΔCD4^ mice affected humoral responses. While at the limit of detection in *Atg16l1*^fl/fl^ controls, serum IgE concentrations were significantly elevated in young *Atg16l1*^ΔCD4^ mice and increased further as the mice aged (Figure 3A). Furthermore, levels of serum IgA and IgG_1_ in young *Atg16l1*^ΔCD4^ mice were also significantly elevated relative to *Atg16l1*^fl/fl^ littermates (Figure 3—figure supplement 1A) and again increased as the *Atg16l1*^ΔCD4^ mice aged (Figure 3B). In contrast, levels of isotypes not associated with T_H_2 help were identical in aged *Atg16l1*^ΔCD4^ mice and *Atg16l1*^fl/fl^ littermates (Figure 3B). Thus, there was a progressive dysregulation of T_H_2-associated antibody responses in *Atg16l1*^ΔCD4^ mice. Consistent with these elevated humoral responses, young *Atg16l1*^ΔCD4^ mice had higher frequencies of germinal center (GC), memory B cells and plasma cells in the spleen and mLN compared to *Atg16l1*^fl/fl^ littermates (Figure 3—figure supplement 1B) and markedly enlarged Peyer’s patches were observed in aged *Atg16l1*^ΔCD4^ mice (Figure 3C).

**Figure 3—figure supplement 1.**
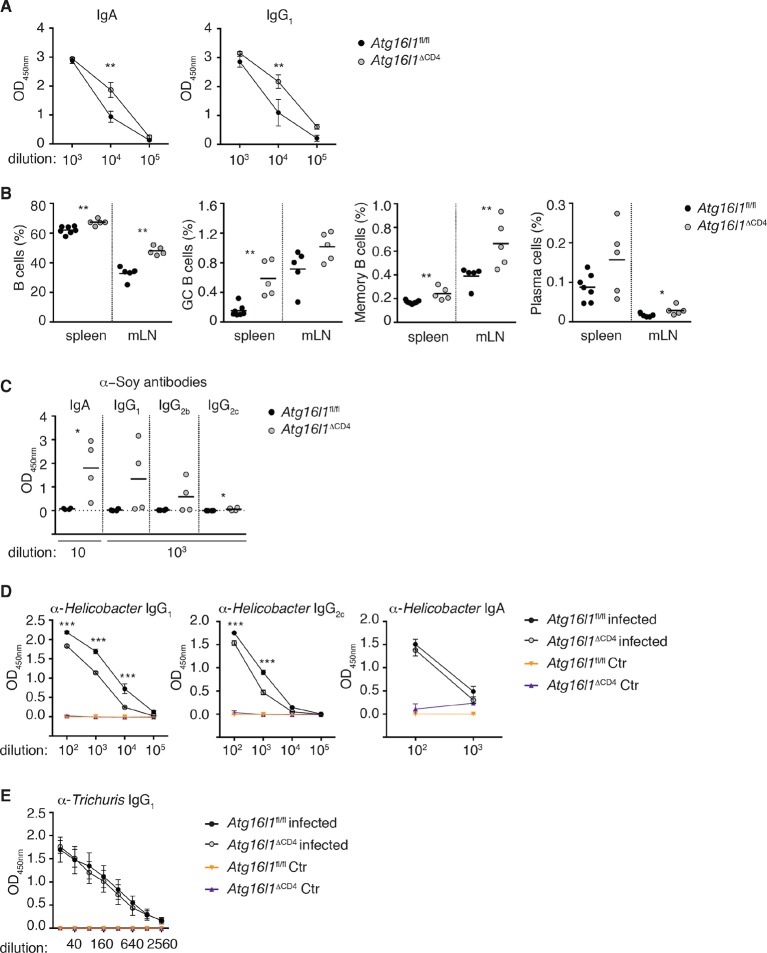
Dysregulated humoral responses in young *Atg16l1*^ΔCD4^ mice. (**A**) Serum antibody IgA and IgG_1_ isotype levels in young *Atg16l1*^ΔCD4^ and *Atg16l1*^fl/fl^ littermates were measured by ELISA. (**B**) Frequencies of B cells (B220^+^), germinal center B cells (GC: B220^+^ GL7^+^ CD95^+^), memory B cells (B220^+^ Gl7^-^ IgM^-^ IgG^+^) and plasma cells (CD138^+^) in the spleen and mLN of young *Atg16l1*^ΔCD4^ and *Atg16l1*^fl/fl^ littermates (gated on live CD45^+^ cells). (**C**) Serum levels of Soy-specific IgA, IgG_1_, IgG_2b_, IgG_2c_ antibodies in young *Atg16l1*^ΔCD4^ and *Atg16l1*^fl/fl^ littermates were measured by ELISA. (**D**) Cohorts of young *Atg16l1*^ΔCD4^ and *Atg16l1*^fl/fl^ littermates were infected with *Helicobacter hepaticus* by oral gavage (three feeds of 1x10^8^ CFU) and levels of serum *Helicobacter*-specific IgG_1_, IgG_2c_ and IgA antibodies were determined three weeks later by ELISA. (**E**) Cohorts of young *Atg16l1*^ΔCD4^ and *Atg16l1*^fl/fl^ littermates were orally infected with *Trichuris muris* (200 eggs) and levels of *T. muris*-specific IgG_1_ in serum were determined 34 days later by ELISA. Data are from one experiment with at least three mice per group (**A-D**) or representative from two independent experiments with five mice per group (**E**). Each dot represents an individual mouse and horizontal bars denote means (**B,C**) or data represent mean ± s.e.m (**A,D,E**). Statistical significance was determined using the Mann–Whitney test (**A,C**) or two-way analysis of variance (ANOVA) with Bonferroni’s correction for multiple comparisons (**B,D,E**): statistical significance was determined between infected groups (b,c), *p<0.05; **p<0.01. mLN - mesenteric lymph nodes. Young mice: 8–12 weeks old. **DOI:** http://dx.doi.org/10.7554/eLife.12444.010

Multiple studies have demonstrated the critical role played by Foxp3^+^ T_reg_ cells in immune tolerance to dietary and microbial antigens within the intestine. Furthermore, changes in intestinal T_reg_ and T_H_2 responses are associated with food hypersensitivities (Berin and Sampson, 2013). We hypothesized that the aberrant humoral responses in *Atg16l1*^ΔCD4^ mice might be directed against luminal antigens. Soy is the main protein source in chow, and we detected high levels of anti-soy IgG_1_ and IgA in sera from aged *Atg16l1*^ΔCD4^ mice, whereas these responses were undetectable in control *Atg16l1*^fl/fl^ littermates (Figure 3D). By contrast, we only detected marginal levels of soy-specific IgG_2b_ or IgG_2c_ in aged *Atg16l1*^ΔCD4^ sera (Figure 3D). Importantly, elevated anti-soy IgG_1_ and IgA antibodies were already present in sera from young *Atg16l1*^ΔCD4^ mice, before the onset of intestinal inflammation (Figure 3—figure supplement 1C). Despite the very high levels of total serum IgE in aged *Atg16l1*^ΔCD4^ mice, we did not detect elevated levels of anti-soy IgE (data not shown). The absence of soy-specific IgE could be due to the inhibiting effects of persistent exposure to high-dose antigens on IgE responses (Sudowe et al., 1997; Riedl et al., 2005). Therefore, to test whether an IgE response was mounted during transient exposure to low-dose dietary antigens, we fed young *Atg16l1*^ΔCD4^ and *Atg16l1*^fl/fl^ mice with ovalbumin (OVA), either alone or in combination with the mucosal adjuvant cholera toxin (CT). As expected, anti-OVA IgE responses were undetectable in control *Atg16l1*^fl/fl^ mice fed OVA alone and were only marginally increased by co-administration of CT (Figure 3E). In contrast, *Atg16l1*^ΔCD4^ mice exhibited significantly elevated levels of anti-OVA IgE after being fed OVA alone and developed >10-fold higher levels of OVA-specific IgE after feeding of OVA with CT (Figure 3E). Together, these results indicate that *Atg16l1*^ΔCD4^ mice displayed aberrant T_H_2-associated antibody responses towards otherwise innocuous dietary protein antigens.

Besides food antigens, the intestinal lumen harbors vast quantities of commensal-derived antigens. Thus, we measured antibodies directed against the flagellin antigen CBir1, produced by commensal bacteria belonging to *Clostridia* cluster XIVa, as antibodies against flagellin are readily detected in sera of IBD patients (Lodes et al., 2004). We detected significantly higher levels of CBir1-specific IgG_1_ and IgA in the serum of aged *Atg16l1*^ΔCD4^ mice compared to control *Atg16l1*^fl/fl^ littermates, whereas anti-CBir1 IgG_2b_ and IgG_2c_ levels were comparable (Figure 3F). Furthermore, CBir1-specific IgG_1_ and IgA were already detectable in young *Atg16l1*^ΔCD4^ mice (data not shown). In contrast, increased T_H_2 cell-associated antibody responses were not mounted in young *Atg16l1*^ΔCD4^ mice following oral infection either with the Gram-negative bacterium *Helicobacter hepaticus* or with the nematode parasite *Trichuris muris* (Figure 3—figure supplement 1D,E). Taken together, these results indicate that the abnormal T_H_2-associated antibody responses observed in *Atg16l1*^ΔCD4^ mice preceded the development of overt inflammation and were selectively induced towards commensal microbiota and dietary antigens.

### Atg16l1 differentially regulates survival of T_H_2 and T_reg_ cells

**Figure 4.**
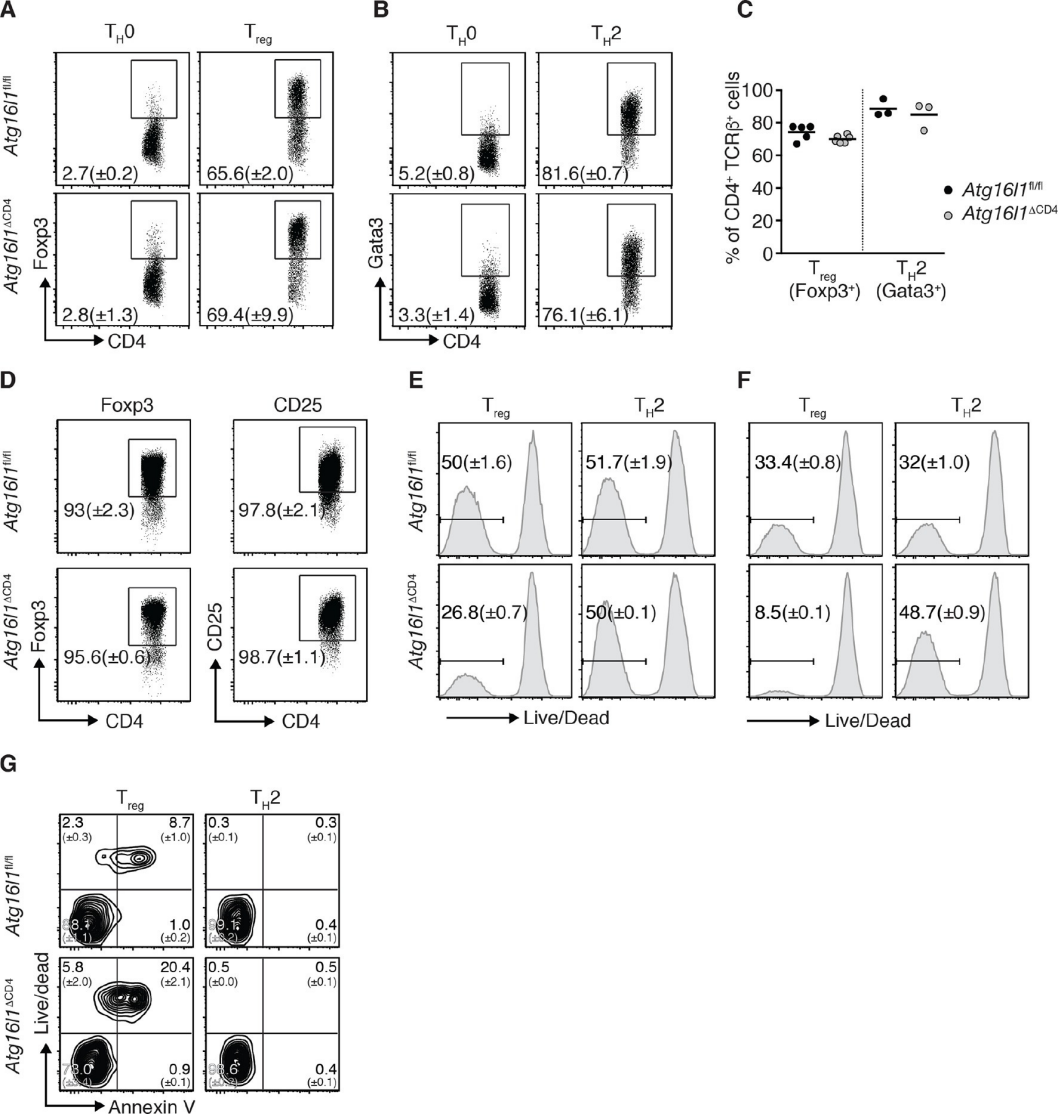
*Atg16l1* promotes survival of T_reg_ cells and limits T_H_2 cell survival. (**A,B**) *Atg16l1*^ΔCD4^ or *Atg16l1*^fl/fl^ naïve CD4^+^ T cells were cultured in T_H_0, T_reg_, or T_H_2 polarizing conditions for 48 hr and analyzed by FACS. Representative FACS plots show (**A**) Foxp3 and (**B**) Gata3 expression (gated on CD4^+^ TCRβ^+^ T cells). (**C**) Frequencies of T_reg_ cells (Foxp3^+^) and T_H_2 cells (Gata3^+^) arising from *Atg16l1*^ΔCD4^ or *Atg16l1*^fl/fl^ naïve CD4^+^ T cells cultured in T_reg_ or T_H_2 polarizing conditions for 5 days. (**D**) *Atg16l1*^ΔCD4^ or *Atg16l1*^fl/fl^ T_reg_ cells were cultured with anti-CD3 (3 μg/ml) and anti-CD28 (1 μg/ml) for 48 hr, then maintained in the presence of IL-4 and IL-13 for a further 5 days before FACS analysis of Foxp3 and CD25 expression of live CD4^+^ T cells. (**E,F**) Naïve *Atg16l1*^ΔCD4^ or *Atg16l1*^fl/fl^ CD4^+^ T cells were cultured with (**E**) 1 μg/ml or (**F**) 5 μg/ml anti-CD3 plus anti-CD28 (1 μg/ml) for 48 hr in T_reg_ or T_H_2 polarizing conditions, then maintained in polarizing conditions for a further 5 days before FACS analysis of cell survival. Histograms show gates and frequencies of live CD4^+^ T cells. (**G**) Representative FACS plots of viability dye and Annexin V staining of T_reg_ cells and T_H_2 cells from the cLP of young *Atg16l1*^ΔCD4^ and *Atg16l1*^fl/fl^ littermates, gated on CD4^+^ TCRβ^+^ Foxp3^+^ (left panel), or CD4^+^ TCRβ^+^ Gata3^+^ (right panel). Data are representative from two (**D,G**) or three independent experiments (**A,B,E,F**), or are combined from three independent experiments (**C**). Each dot represents an individual cell culture (**C**) or data are shown as mean ± s.e.m (**A,B,D-F**). Numbers indicate percentage of cells in quadrants (**G**). cLP – colonic lamina propria. Young mice: 8–12 weeks old. **DOI:** http://dx.doi.org/10.7554/eLife.12444.011

Given apparent opposing effects of *Atg16l1* deficiency on T_H_2 and T_reg_ cells, we questioned whether the disruption of autophagy pathway affects the differentiation of these T cell subsets. We found that, under T_H_2 or T_reg_ polarizing conditions, differentiation of naïve CD4^+^ T cells isolated from *Atg16l1*^ΔCD4^ or *Atg16l1*^fl/fl^ littermates toward the Gata3^+^ T_H_2 or Foxp3^+^ T_reg_ cell phenotype was comparable (Figure 4A–C). As T_H_2 cytokines can negatively affect T_reg_ differentiation and stability (Dardalhon et al., 2008; Feng et al., 2014), it was possible that outgrowth of T_H_2 cells may also have contributed to the loss of intestinal T_reg_ in *Atg16l1*^ΔCD4^ mice. We therefore isolated Foxp3^+^ T_reg_ cells from *Atg16l1*^ΔCD4^ and *Atg16l1*^fl/fl^ littermates and activated them in vitro in the presence of IL-4 and IL-13. However, we did not find any evidence of T_reg_ instability, as expression of Foxp3 and CD25 remained equally high in *Atg16l1*-deficient and WT T_reg_ cells (Figure 4D).

We therefore examined whether autophagy deficiency influenced the survival of T_H_2 or Foxp3^+^ T_reg_ cells. Thus, naïve CD4^+^ T cells isolated from *Atg16l1*^ΔCD4^ or *Atg16l1*^fl/fl^ littermates were activated for 48 hr with anti-CD3 and anti-CD28 antibodies and then rested for 5 days. Cells were kept in T_H_2 or T_reg_ polarizing conditions throughout the experiment. Following activation with different concentrations of anti-CD3 antibody, *Atg16l1*-deficient T_H_2 cells exhibited comparable or improved survival relative to WT T_H_2 cells (Figure 4E,F). In contrast, there was a 50–75% decrease in survival of *Atg16l1*-deficient T_reg_ cells when compared to *Atg16l1*-sufficient T_reg_ cells activated under the same conditions (Figure 4E,F). To establish whether autophagy-deficient T_reg_ and T_H_2 cells exhibited similarly distinct survival profiles in vivo CD4^+^ T cells isolated from cLP of *Atg16l1*^ΔCD4^ or *Atg16l1*^fl/fl^ littermates were stained with a viability dye and Annexin V. We observed that an increased proportion of *Atg16l1*-deficient intestinal T_reg_ cells were dead or dying compared to WT T_reg_ cells (Figure 4G). In contrast, *Atg16l1*-deficiency had no negative effect on the viability of intestinal T_H_2 cells, which was comparable to WT controls (Figure 4G). Together, these results indicate that *Atg16l1*-deficiency does not impair the differentiation or stability of T_reg_ cells and does not promote differentiation towards the T_H_2 lineage. However, autophagy differentially impacts on the survival of mucosal T_reg_ and T_H_2 cells.

### Autophagy regulates intestinal T_H_2 responses in a cell-intrinsic manner

**Figure 5.**
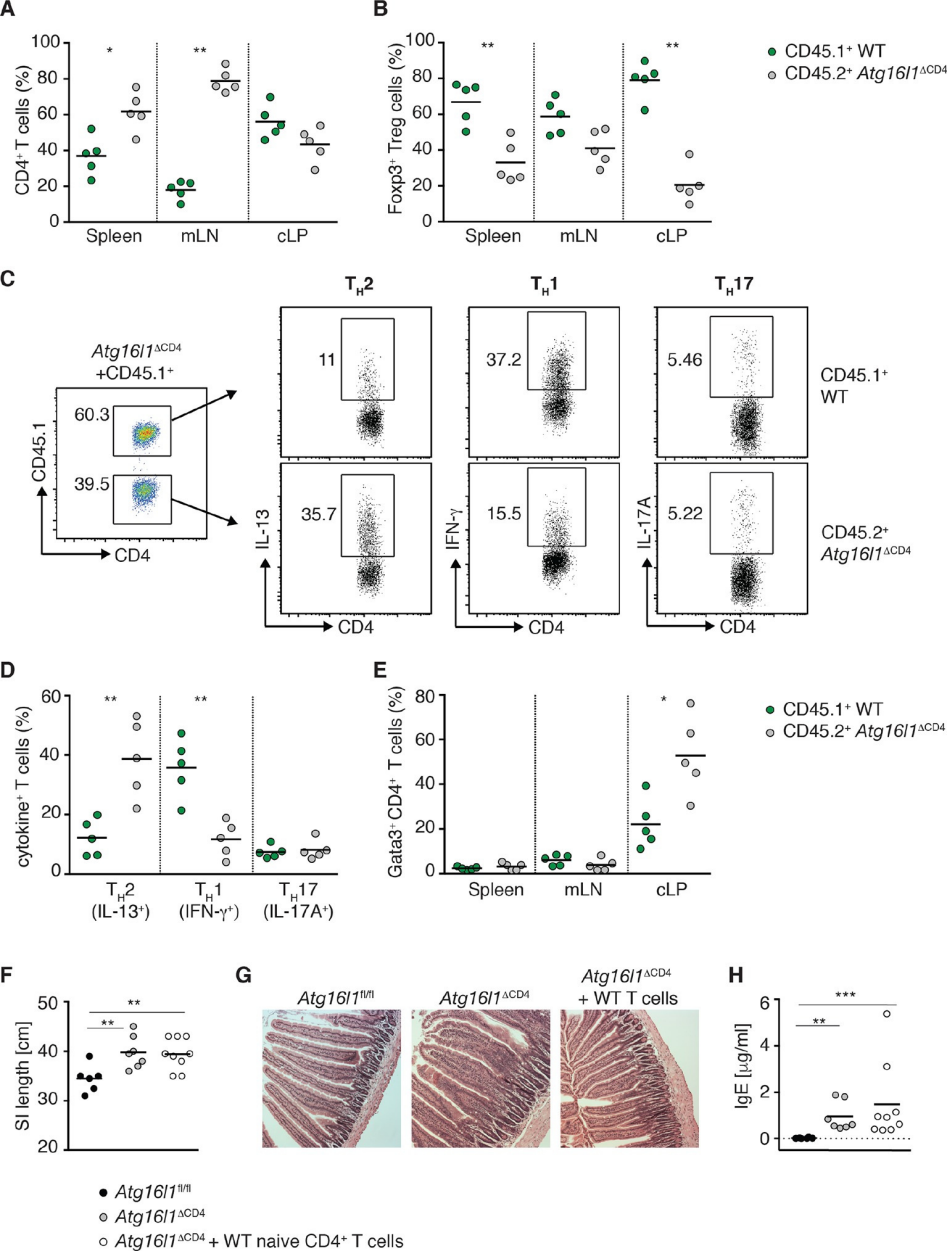
Autophagy contributes to the elevated T_H_2 responses in *Atg16l1*^ΔCD4^ mice in a cell-intrinsic manner. Young *Atg16l1*^ΔCD4^ mice (CD45.2^+^) were adoptively transferred with 4-5x10^6^ naïve WT CD4^+^ T cells (CD45.1^+^) and analyzed 3 months later. (**A**) Frequencies of WT (CD45.1^+^) and *Atg16l1-*deficient (CD45.2^+^) CD4^+^ T cells in the spleen, mLN and cLP. (**B**) Frequencies of WT (CD45.1^+^) and *Atg16l1-*deficient (CD45.2^+^) Foxp3^+^ T_reg_ cells in the spleen, mLN and cLP (gated on CD4^+^ TCRβ^+^ T cells). (**C**) Representative FACS plots showing gating of WT (CD45.1^+^) and *Atg16l1-*deficient (CD45.1^-^) CD4^+^ T cells and expression of IL-13 (T_H_2), IFN-γ (T_H_1) and IL-17A (T_H_17) in the cLP (gated on CD4^+^ TCRβ^+^ Foxp3^-^ T cells). (**D**) Frequencies of WT (CD45.1^+^) and *Atg16l1-*deficient (CD45.2^+^) T_H_2 (IL-13^+^), T_H_1 (IFN- γ^+^) and T_H_17 (IL-17A^+^) cells among CD4^+^ TCRβ^+^ Foxp3^-^ T cells in the cLP. (**E**) Frequencies of WT (CD45.1^+^) and *Atg16l1-*deficient (CD45.2^+^) Gata3^+^ CD4^+^ T cells in the spleen, mLN and cLP (gated on CD4^+^ TCRβ^+^ Foxp3^-^ T cells). (**F**) SI lengths and (**G**) representative photomicrographs of jejunum of control untreated *Atg16l1*^fl/fl^ or *Atg16l1*^ΔCD4^ littermates and reconstituted *Atg16l1*^ΔCD4^ mice, scale bar 150 μm. (**H**) Serum IgE concentrations in control untreated *Atg16l1*^fl/fl^ or *Atg16l1*^ΔCD4^ littermates and adoptively transferred *Atg16l1*^ΔCD4^ mice were measured by ELISA. Data are representative of two independent experiments with at least four mice per group (**A-E,G**) or combined from two independent experiments (**F,H**). Each dot represents cells coming from the donor or the hosts within the individual transferred mouse (**A,B,D,E**) or each dot represents an individual mouse (**F,H**), horizontal bars denote mean. Numbers indicate percentage of cells in gates. Statistical significance was determined using the Mann–Whitney test, *p<0.05; **p<0.01. mLN - mesenteric lymph nodes, cLP – colonic lamina propria. Young mice: 10–12 weeks old. **DOI:** http://dx.doi.org/10.7554/eLife.12444.012

As pT_reg_ cells are required to control T_H_2 responses at mucosal sites (Mucida et al., 2005; Curotto de Lafaille et al., 2008; Josefowicz et al., 2012), we examined whether the enhanced T_H_2 phenotype in *Atg16l1*^ΔCD4^ mice could be corrected by reconstitution of the intestinal Foxp3^+^ T_reg_ compartment. We restored the pTreg population in young *Atg16l1*^ΔCD4^ mice at the age of 10–12 weeks, before the onset of intestinal pathology, through adoptive transfer of congenic WT naïve CD45.1^+^ CD4^+^ T cells. Recipients were sacrificed 3 months later, when control *Atg16l1*^ΔCD4^ littermates had developed intestinal inflammation. We detected CD45.1^+^ donor CD4^+^ T cells in all adoptively transferred *Atg16l1*^ΔCD4^ mice, but the level of reconstitution varied by the organ examined. In reconstituted *Atg16l1*^ΔCD4^ mice, donor WT CD4^+^ T cells accounted for 37 ± 5% of total CD4^+^ T cells in spleen and 18 ± 2% in mLN, whereas in the cLP they represented 56 ± 4% (Figure 5A). Thus, autophagy-deficient CD4^+^ T cells had a survival disadvantage when compared to WT CD4^+^ T cells within the intestinal mucosa. Overall, adoptive transfer of WT naïve CD4^+^ T cells restored the total frequencies of CD4^+^ T cells in the cLP to levels comparable to control *Atg16l1*^fl/fl^ mice (Figure 5—figure supplement 1A).

**Figure 5—figure supplement 1.**
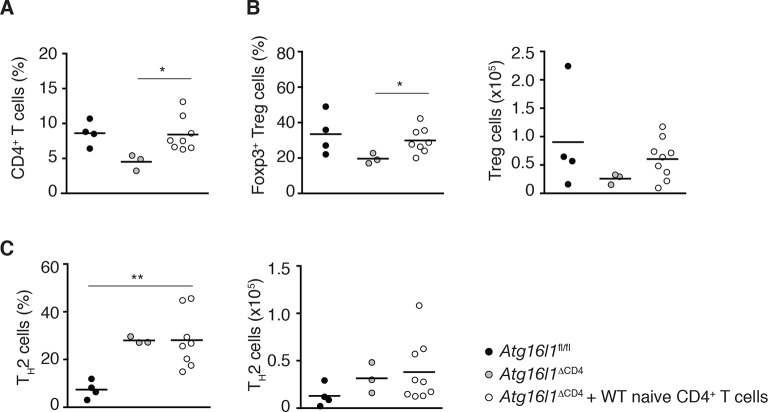
Reconstitution of intestinal CD4^+^ T cell compartments in adoptively transferred *Atg16l1*^ΔCD4^ mice. Young *Atg16l1*^ΔCD4^ mice (CD45.2^+^) were adoptively transferred with 4-5x10^6^ naïve WT CD4^+^ T cells (CD45.1^+^) *i.v.* and cLP populations analyzed 3 months later. (**A**) Frequencies of total CD4^+^ T cells. (**B**) Frequencies and total numbers of Foxp3^+^ T_reg_ cells (gated on CD4^+^ TCRβ^+^ T cells). (**C**) Frequencies and total numbers of T_H_2 (IL-13^+^) cells (gated on CD4^+^ TCRβ^+^ Foxp3^-^ T cells). Data are combined from two independent experiments with at least four mice per group. Each dot represents an individual mouse, horizontal bars denote mean. Statistical significance was determined using the Mann–Whitney test, *p<0.05; **p<0.01. cLP – colonic lamina propria. Young mice: 10–12 weeks old. **DOI:** http://dx.doi.org/10.7554/eLife.12444.013

When we examined Foxp3^+^ T_reg_ cells, the survival advantage conferred by autophagy was even more apparent, with around 50% of the donor WT naïve CD45.1^+^ T cells developing into Foxp3^+^ pT_reg_ cells in spleen, mLN and cLP of *Atg16l1*^ΔCD4^ recipients. As a result, the majority of Foxp3^+^ T_reg_ cells were of WT donor origin (67 ± 5% in spleen, 59 ± 4% in mLN and 80 ± 5% in cLP) (Figure 5B). Thus, adoptive transfer of WT-naïve CD4^+^ T cells resulted in efficient reconstitution of Foxp3^+^ pT_reg_ cells in *Atg16l1*^ΔCD4^ mice; the total frequencies and numbers of T_reg_ cells within the cLP of transferred mice were comparable with control *Atg16l1*^fl/fl^ mice (Figure 5—figure supplement 1B). As such, we could utilize this system to determine whether excessive T_H_2 cell accumulation in *Atg16l1*^ΔCD4^ mice was due to impaired mucosal pT_reg_ cells or to a cell-intrinsic effect of *Atg16l1*-deficiency in T_H_2 cells.

When we analyzed the frequencies of T_H_2 cells in the cLP of reconstituted *Atg16l1*^ΔCD4^ mice, we observed significantly higher frequencies of Gata3^+^ IL-13^+^ T_H_2 cells among *Atg16l1*-deficient CD45.2^+^ CD4^+^ T cells compared with the WT donor CD45.1^+^ CD4^+^ T cells (Figure 5C–E). Indeed, frequencies of IL-13^+ ^*Atg16l1*-deficient CD4^+^ T cells in the cLP of pT_reg_-reconstituted mice were comparable to those found in untreated *Atg16l1*^ΔCD4^ littermates (Figure 5C,D). In contrast, there was no difference in T_H_17 cell frequencies between *Atg16l1*-deficient CD45.2^+^ and WT CD4^+^ T cells, and there was a significant decrease in T_H_1 frequencies among *Atg16l1*-deficient CD4^+^ T cells (Figure 5C,D). In line with these observations, adoptively transferred *Atg16l1*^ΔCD4^ mice had comparable total frequencies and numbers of T_H_2 cells as observed in untreated *Atg16l1*^ΔCD4^ mice (Figure 5—figure supplement 1C). Thus, provision of WT pT_reg_ cells did not rescue the increased T_H_2 phenotype of *Atg16l1*-deficient CD4^+^ T cells, indicating that autophagy directly regulates T_H_2 cells through a cell-intrinsic mechanism. Consistent with this finding, *Atg16l1*^ΔCD4^ mice reconstituted with WT pT_reg_ cells still developed intestinal pathology and elevated serum IgE levels comparable to those present in untreated *Atg16l1*^ΔCD4^ littermates (Figure 5F–H).

### Autophagy is essential for T_reg_ cell homeostasis and control of effector T cell responses in the gut

**Figure 6.**
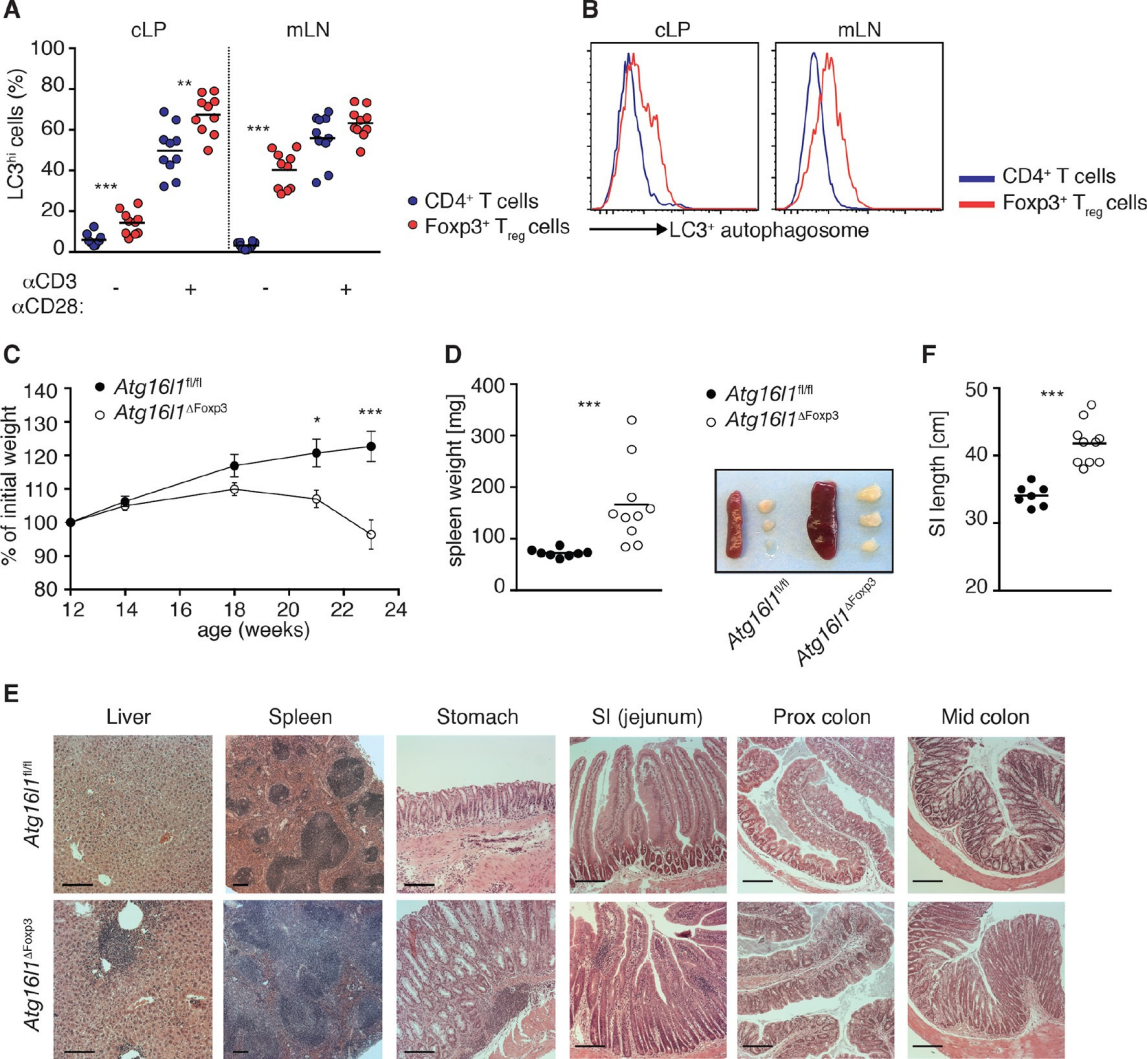
Aged *Atg16l1*^ΔFoxp3^ mice develop spontaneous multi-organ inflammation. (**A**) LC3^+^ autophagosome formation by Foxp3^-^ CD4^+^ T cells and Foxp3^+^ T_reg_ cells from cLP and mLN of WT mice in unstimulated cells or after overnight activation with α-CD3 (5 μg/ml) and α-CD28 (1 μg/ml). (**B**) Representative LC3 staining of unstimulated cells (gated on Foxp3^+^ CD4^+^ TCRβ^+^ T_reg_ cells or Foxp3^-^ CD4^+^ TCRβ^+^ T cells). (**C**) Weight curves and (**D**) spleen weights and representative images of spleen and mLN of aged *Atg16l1*^ΔFoxp3^ and *Atg16l1*^fl/fl^ littermates. (**E**) Representative photomicrographs of H&E stained sections of liver, spleen, stomach, SI (jejunum), proximal colon and mid-colon of aged *Atg16l1*^ΔFoxp3^ and *Atg16l1*^fl/fl^ littermates, scale bar 150 μm. (**F**) Quantification of SI length. Data are combined from two to four independent experiments with two to five mice per group (**A**,**D**,**F**) or are representative of two to three independent experiments with two to five mice per group (**B**,**C**,**E**). Each dot represents an individual mouse and horizontal bars denote means (**A**,**D**,**F**). Data shown as mean ± s.e.m (**C**). Statistical significance was determined using two-way analysis of variance (ANOVA) with Bonferroni’s correction for multiple comparisons (**C**) or using the Mann–Whitney test (**A**,**D**,**F**), *p<0.05; **p<0.01; ***p<0.001. mLN - mesenteric lymph nodes, SI – small intestine lamina propria, cLP – colonic lamina propria. Aged mice >5 months old. **DOI:** http://dx.doi.org/10.7554/eLife.12444.014

Given that *Atg16l1*-deficiency significantly reduced the number of intestinal T_reg_ cells in *Atg16l1*^ΔCD4^ mice, we hypothesized that T_reg_ cells may be particularly reliant on autophagy compared to other subsets of CD4^+^ T cells. Indeed, in WT mice we found that levels of autophagy were significantly higher in Foxp3^+^ T_reg_ cells compared to Foxp3^-^ CD4^+^ T cells, both constitutively and after TCR activation (Figure 6A,B). Together with our observations of impaired survival of *Atg16l1*-deficient Foxp3^+^ T_reg_ cells (Figure 4E–G), this suggested an important cell-intrinsic role for autophagy in the maintenance of T_reg_ cells. This hypothesis was further strengthened by analyses of mixed bone marrow (BM) chimeras where irradiated *Rag1*^-/-^ mice were reconstituted with a 1:1 mixture of BM cells from *Atg16l1*^ΔCD4^ mice and congenic WT C57BL/6mice (Figure 6—figure supplement 1A). In this setting, the reconstitution of CD4^+^ T cells was severely hampered in the absence of functional autophagy and this deficiency was most pronounced in the T_reg_ compartment of the spleen and cLP (Figure 6—figure supplement 1B–E), confirming that *Atg16l1*-deficiency decreases the ability of Foxp3^+^ T_reg_ cells to compete with WT T_reg_ cells in a cell-intrinsic manner.

**Figure 6—figure supplement 1.**
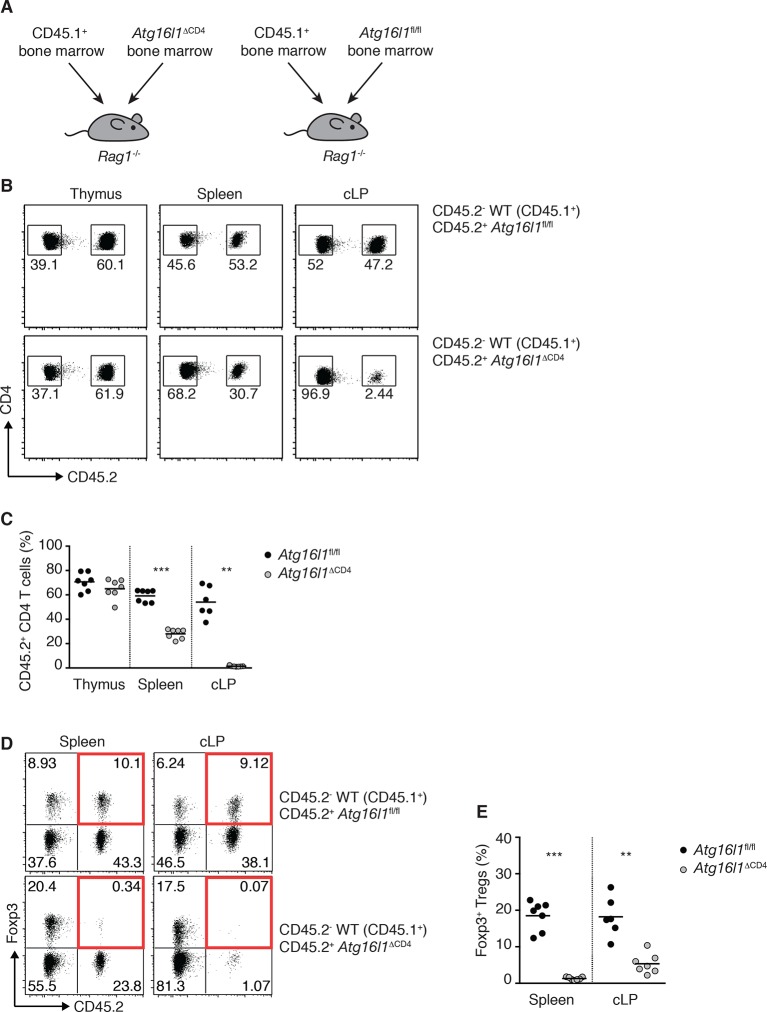
Impaired reconstitution of mixed bone marrow chimeras by *Atg16l1*-deficient T cells. (**A**) Experimental design for the generation of mixed bone marrow (BM) chimeras. BM cells isolated from WT (CD45.1^+^) and *Atg16l1*^fl/fl^ or *Atg16l1*^ΔCD4^ (CD45.2^+^) mice were injected at a 1:1 ratio into lethally irradiated (1100 Rad) *Rag1*^-/-^ recipients (total of 1x10^7^ cells per mouse). (**B**) Representative FACS plots showing frequencies of *Atg16l1*^ΔCD4^ or *Atg16l1*^fl/fl^ (CD45.2^+^) and WT (CD45.2^-^) CD4^+^ T cells in the thymus, spleen and cLP of mixed BM chimeras (gated on CD4^+^ TCRβ^+^ T cells). (**C**) Frequencies of *Atg16l1*^ΔCD4^ or *Atg16l1*^fl/fl^ (CD45.2^+^) CD4^+^ T cells in mixed BM chimeras (shown as percentage of total CD4^+^ TCRβ^+^ T cells). (**D**) Representative FACS plots and (**E**) frequencies of Foxp3^+^ T_reg_ cells derived from *Atg16l1*^ΔCD4^ or *Atg16l1*^fl/fl^ (CD45.2^+^) cells in mixed BM chimeras (gated on CD4^+^ TCRβ^+^ T cells). Highlighted top right quadrants indicate T_reg_ cells derived from *Atg16l1*^fl/fl^ or*Atg16l1*^ΔCD4^ BM. Data are representative from two independent experiments with at least seven mice per group. Each dot represents an individual mouse and horizontal bars denote means. Numbers indicate percentage of cells in gates. Statistical significance was determined using the Mann–Whitney test, **p<0.01; ***p<0.001. **DOI:** http://dx.doi.org/10.7554/eLife.12444.015

**Figure 6—figure supplement 2.**
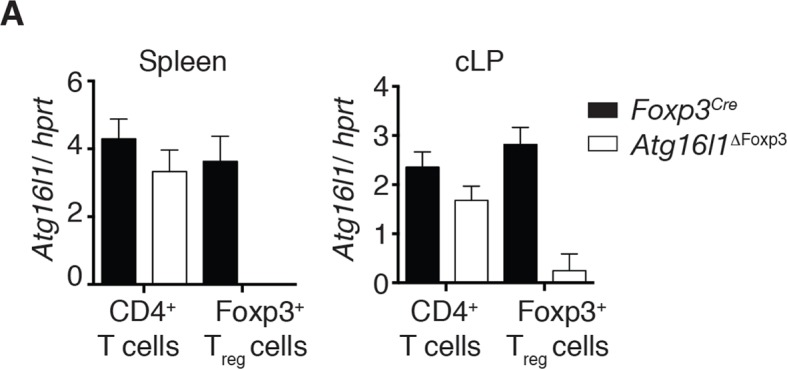
Analysis of *Atg16l1* expression in *Atg16l1*^ΔFoxp3^ mice. (**A**) qPCR analysis of *Atg16l1* exon 3 levels in sorted CD4^+^ Foxp3^-^ T cells and Foxp3^+^ T_reg_ cells from spleen and cLP of *Atg16l1*^ΔFoxp3^ and *Foxp3*^Cre^ mice. Data are representative from two independent experiments with 5 mice per group. *Atg16l1* exon 3 levels are shown as mean ± s.e.m of three technical replicates, normalised to expression of *hprt*. cLP – colonic lamina propria. **DOI:** http://dx.doi.org/10.7554/eLife.12444.016

**Figure 7.**
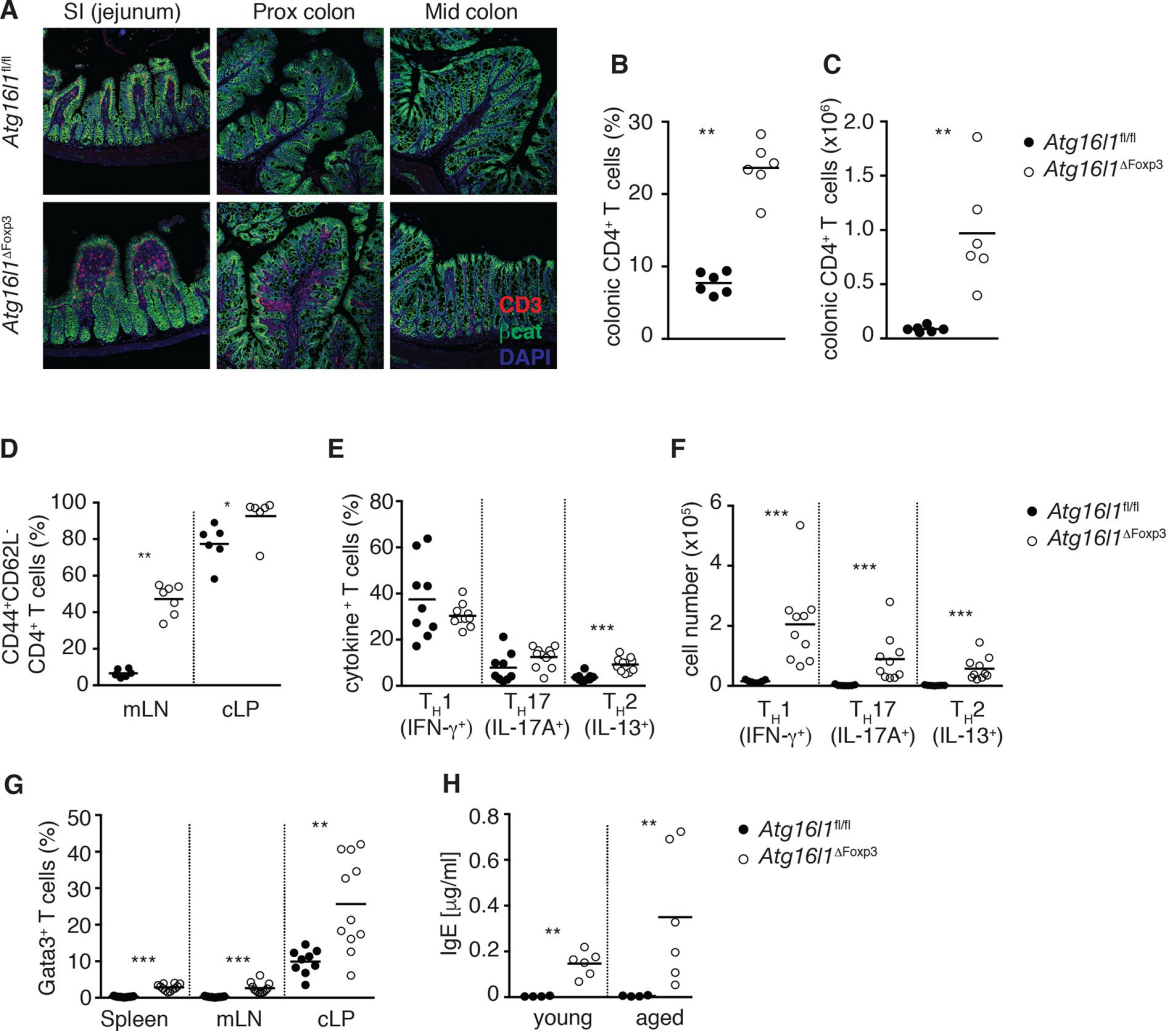
*Atg16l1*^ΔFoxp3^ mice cannot control pro-inflammatory T_H_ effector responses. (**A**) Representative immunofluorescence images of small intestine and proximal and mid colon of aged *Atg16l1*^ΔFoxp3^ and *Atg16l1*^fl/fl^ littermates stained for CD3 (red), β-catenin (green) and DAPI (blue). (**B**) Frequencies and (**C**) total numbers of cLP CD4^+^ TCRβ^+^ T cells in aged *Atg16l1*^ΔFoxp3^ and *Atg16l1*^fl/fl^ littermates. (**D**) Frequencies of effector (CD44^+^CD62L^-^) CD4^+^ T cells in the mLN and cLP of aged *Atg16l1*^ΔFoxp3^ and *Atg16l1*^fl/fl^ littermates (gated on CD4^+^ TCRβ^+^ Foxp3^-^ T cells). (**E**) Frequencies and (**F**) total numbers of T_H_1 (IFN-γ^+^), T_H_17 (IL-17A^+^), T_H_2 (IL-13^+^) T cells in the cLP of aged *Atg16l1*^ΔFoxp3^ and *Atg16l1*^fl/fl^ littermates (gated on CD4^+^ TCRβ^+^ Foxp3^-^ T cells). (**G**) Frequencies of Gata3^+^ CD4^+^ T cells in aged *Atg16l1*^ΔFoxp3^ and *Atg16l1*^fl/fl^ littermates (gated on CD4^+^ TCRβ^+^ Foxp3^-^ T cells). (**H**) Serum IgE concentrations in *Atg16l1*^ΔFoxp3^ and *Atg16l1*^fl/fl^ littermates were measured by ELISA. Data are combined from two to four independent experiments with two to five mice per group (**B-H**) or are representative of two independent experiments with two to five mice per group (**A**). Each dot represents an individual mouse and horizontal bars denote means. Statistical significance was determined using the Mann–Whitney test *p<0.05; **p<0.01; ***p<0.001. mLN - mesenteric lymph nodes, cLP – colonic lamina propria. Young mice: 8–12 weeks old, aged mice >5 months old. **DOI:** http://dx.doi.org/10.7554/eLife.12444.017

To definitively assess the cell-intrinsic requirement for autophagy in Foxp3^+^ T_reg_ cells we crossed *Atg16l1*^fl/fl^ mice with mice expressing a YFP-Cre from the *Foxp3* locus (*Foxp3*^Cre^ mice) (Rubtsov et al., 2008), generating *Atg16l1*^fl/fl^::*Foxp3*^Cre^ mice (hereafter denoted as *Atg16l1*^ΔFoxp3^) in which *Atg16l1* is selectively ablated in Foxp3^+^ T_reg_ cells. These mice allowed us to analyze the consequences of a lack of autophagy in T_reg_ cells in the context of autophagy-competent CD4^+^ T effector cells. As expected, *Atg16l1*^ΔFoxp3^ mice showed a significant reduction of *Atg16l1* expression in Foxp3^+^ T_reg_ cells, but not in CD4^+^ Foxp3^-^ T cells (Figure 6—figure supplement 2A). Although *Atg16l1*^ΔFoxp3^ mice appeared normal in early life, at around 5 months of age they developed a severe spontaneous inflammatory disease characterized by progressive weight loss, splenomegaly, lymphadenopathy and leukocyte infiltration in multiple tissues (Figure 6C–E). The gastrointestinal tract was particularly affected in aged *Atg16l1*^ΔFoxp3^ mice, with marked inflammation in the SI and colon (Figure 6E,F). Intestinal inflammation in aged *Atg16l1*^ΔFoxp3^ mice was characterized by massive accumulation of activated CD4^+^ T cells in the intestinal LP and mLN (Figure 7A–D and Figure 7—figure supplement 1A). The cLP infiltrate in aged *Atg16l1*^ΔFoxp3^ mice contained a mixed population of T_H_1, T_H_17 and T_H_2 effector cells, with a significant increase in the frequencies of IL-13^+^ CD4^+^ T_H_2 cells (Figure 7E,F), although this T_H_2 bias was not present in young *Atg16l1*^ΔFoxp3^ mice (Figure 7—figure supplement 1B). In addition, we observed increased frequencies of Gata3^+^ CD4^+^ T cells in the spleen, mLN and cLP of aged *Atg16l1*^ΔFoxp3^ mice (Figure 7G). Analyses of humoral responses in aged *Atg16l1*^ΔFoxp3^ mice revealed significantly elevated levels of circulating IgE and IgA, however IgG_1_ levels were not increased (Figure 7H and Figure 7—figure supplement 1C). Thus, selective ablation of *Atg16l1* in Foxp3^+^ T_reg_ cells led to intestinal inflammation that was characterized by accumulation of all T_H_ effector types, with a disproportionate increase in T_H_2 responses in aged mice. However, the breadth and magnitude of T_H_2-associated responses were less pronounced in *Atg16l1*^ΔFoxp3^ mice compared to those observed in *Atg16l1*^ΔCD4^﻿ mice.

**Figure 7—figure supplement 1.**
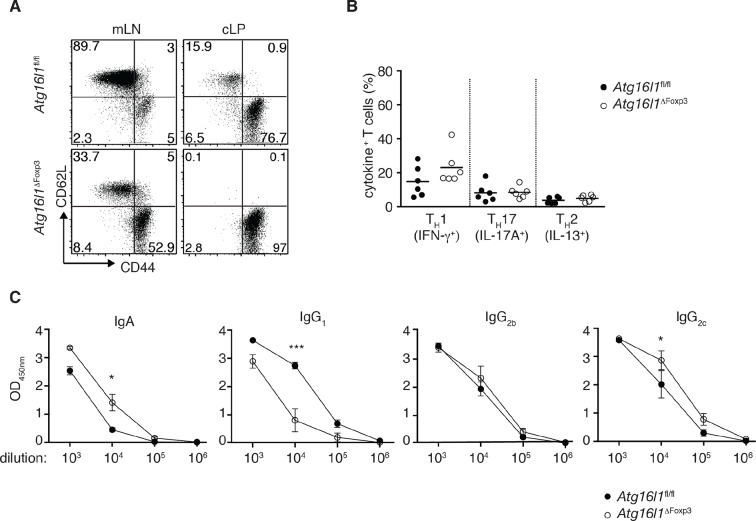
Additional characterization of *Atg16l1*^ΔFoxp3^ mice. (**A**) Representative FACS plots of CD44 and CD62L expression by CD4^+^ T cells from cLP of aged *Atg16l1*^ΔFoxp3^ and *Atg16l1*^fl/fl^ littermates (gated on CD4^+^ TCRβ^+^ Foxp3^-^ T cells). (**B**) Frequencies of T_H_1 (IFN-γ^+^), T_H_17 (IL-17A^+^), T_H_2 (IL-13^+^) T cells in the cLP of young *Atg16l1*^ΔFoxp3^ and *Atg16l1*^fl/fl^ littermates (gated on CD4^+^ TCRβ^+^ Foxp3^-^ T cells). (**C**) Serum antibody IgA, IgG_1_, IgG_2b_, IgG_2c_ isotype levels in aged *Atg16l1*^ΔFoxp3^ and *Atg16l1*^fl/fl^ littermates. Data are combined from three independent experiments with two to five mice per group (**B**) or are representative from two to three independent experiments with two to five mice per group (**A,C**). Each dot represents an individual mouse and horizontal bars denote means. Numbers indicate percentage of cells in gates. Data shown as mean ± s.e.m (**C**). Statistical significance was determined using the Mann–Whitney test, *p<0.05; **p<0.01; ***p<0.001. mLN - mesenteric lymph nodes, cLP – colonic lamina propria. Young mice: 8–12 weeks old, aged mice >5 months old. **DOI:** http://dx.doi.org/10.7554/eLife.12444.018

**Figure 8.**
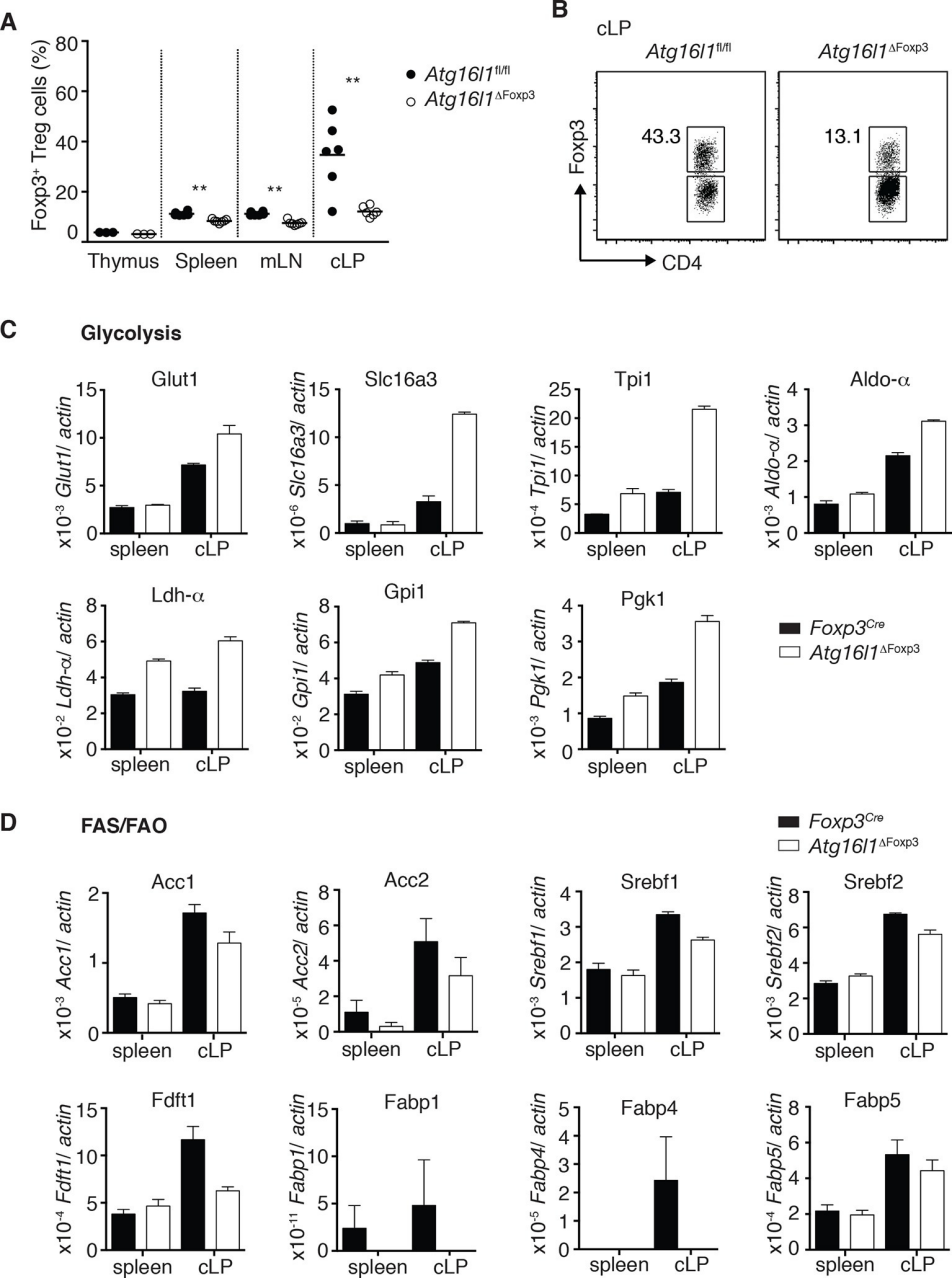
Cell-intrinsic autophagy is required for metabolic adaptation and survival of intestinal Foxp3^+^ T_reg_ cells. (**A**) Foxp3^+^ T_reg_ cell frequencies among CD4^+^ TCRβ^+^ T cells in *Atg16l1*^ΔFoxp3^ and *Atg16l1*^fl/fl^ littermates and (**B**) representative FACS plots of Foxp3 expression in cLP CD4^+^ T cells from young *Atg16l1*^ΔFoxp3^ and *Atg16l1*^fl/fl^ littermates (gated on CD4^+^ TCRβ^+^ T cells). (**C**) qPCR analysis of glycolytic gene levels in sorted Foxp3^+^ T_reg_ cells from spleen and cLP of young *Atg16l1*^ΔFoxp3^ and *Foxp3*^Cre^ mice (sorted for CD4^+^ TCRβ^+^ YFP^+^). (**D**) qPCR analysis of FAS and FAO gene levels in Foxp3^+^ T_reg_ cells from the spleen and cLP of young *Atg16l1*^ΔFoxp3^ and *Foxp3*^Cre^ mice (sorted for CD4^+^ TCRβ^+^ YFP^+^). FAS: fatty acid synthesis, FAO: fatty acid oxidation, Glut1: glucose transporter 1, Slc16ac: solute carrier family 16 member 3 (lactic acid and pyruvate transporter), Tpi1: triosephosphate isomerase 1, Aldo–α: aldolase α, Ldh-α: lactate dehydrogenase α, Gpi1: Glucose phosphate isomerase 1, Pgk1: Phosphoglycerate kinase 1, Acc1: acetyl-CoA carboxylase 1, Acc2: acetyl-CoA carboxylase 2, Srebf1: sterol regulatory element binding transcription factor 1, Srebf2: sterol regulatory element binding transcription factor 2, Fdft1: farnesyl-diphosphate farnesyltransferase 1, Fabp: Fatty acid-binding protein. Data are representative from two (**C,D**) or three independent experiments (**A,B**). Each dot represents individual mouse (**A**) or data are shown as mean ± s.e.m (**C,D**). Gene expression levels are shown as mean ± s.e.m of three technical replicates (**C,D**). Numbers indicate percentage of cells in gates (**B**). cLP – colonic lamina propria. Young mice: 8–12 weeks old. **DOI:** http://dx.doi.org/10.7554/eLife.12444.019

When we examined the T_reg_ cell compartment in *Atg16l1*^ΔFoxp3^ mice, we found significantly decreased frequencies of Foxp3^+^ T_reg_ cells in the spleen and mLN compared to *Atg16l1*^fl/fl^ littermates, although thymic T_reg_ cell frequencies were similar (Figure 8A). As found in *Atg16l1*^ΔCD4^ mice, intestinal LP Foxp3^+^ T_reg_ cells were severely depleted in *Atg16l1*^ΔFoxp3^ mice and those remaining exhibited significantly increased expression of effector T_H_ cytokines (Figure 8A,B and Figure 8—figure supplement 1A). Thus, T_reg_ cell-specific deletion of *Atg16l1* recapitulated the T_reg_ cell deficits observed in *Atg16l1*^ΔCD4^ mice, showing that cell-intrinsic autophagy is essential for peripheral T_reg_ cell homeostasis, especially in the intestine.

**Figure 8—figure supplement 1.**
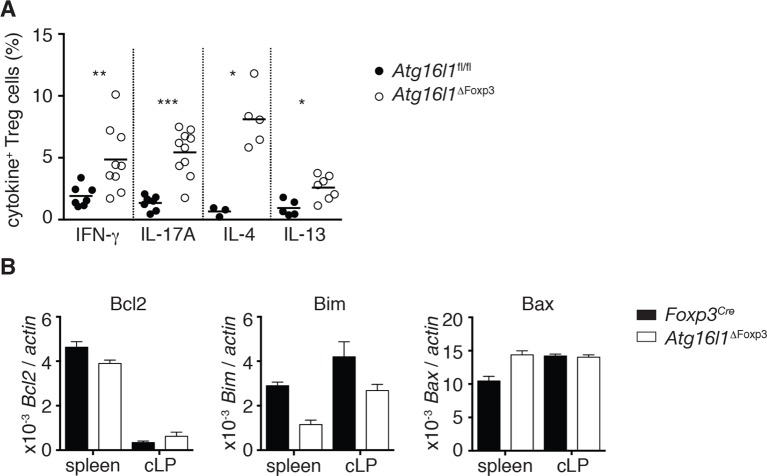
*Atg16l1*-deficient colonic T_reg_ cells exhibit increased cytokine secretion. (**A**) Frequencies of IFN-γ^+^, IL-17A^+^, IL-4^+^, IL-13^+^ Foxp3^+^ T_reg_ cells in the cLP of aged *Atg16l1*^ΔFoxp3^ and *Atg16l1*^fl/fl^ littermates (gated on Foxp3^+^ CD4^+^ TCRβ^+^ T cells). (**B**) qPCR analysis of *Bcl2, Bim* and *Bax* levels in Foxp3^+^ T_reg_ cells from young cLP of *Atg16l1*^ΔFoxp3^ and *Foxp3*^Cre^ mice (sorted for CD4^+^ TCRβ^+^ YFP^+^). Data are combined from three independent experiments with two to five mice per group (**A**) or are representative from two independent experiments (**B**). Each dot represents an individual mouse and horizontal bars denote means (**A**). Gene expression levels are shown as mean ± s.e.m of three technical replicates (**B**). cLP – colonic lamina propria. Young mice: 8–12 weeks old. **DOI:** http://dx.doi.org/10.7554/eLife.12444.020

**Figure 8—figure supplement 2.**
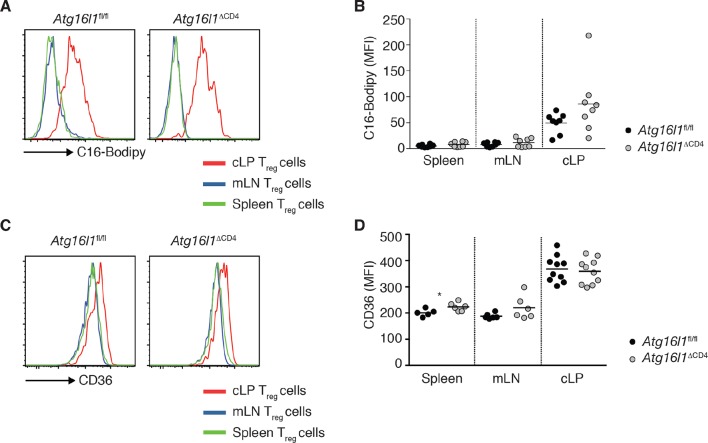
Increased lipid uptake by intestinal T_reg_ cells. (**A,B**) *Atg16l1*^fl/fl^ and *Atg16l1*^ΔCD4^ littermates were injected i.p. with 50 μg of fluorescent 16-carbon fatty acid analog BODIPY C-16 and culled 1 hr later and tissue was collected for analysis by flow cytometry. (**A**) Representative FACS plots and (**B**) quantification of C16-Bodipy uptake by Foxp3^+^ T_reg_ cells in the spleen, mLN and cLP (gated on Foxp3^+^ CD4^+^ TCRβ^+^ T cells). (**C**) Representative FACS plots and (**D**) quantification of CD36 expression by Foxp3^+^ T_reg_ cells in the spleen, mLN and cLP of *Atg16l1*^fl/fl^ and *Atg16l1*^ΔCD4^ littermates (gated on Foxp3^+^ CD4^+^ TCRβ^+^ T cells). Data are combined from (**B,D**) or are representative of (**A,C**) two independent experiments with 3–5 mice per group. Each dot represents an individual mouse and horizontal bars denote means. Statistical significance was determined using the Mann–Whitney test, *p<0.05. mLN - mesenteric lymph nodes, cLP – colonic lamina propria. Young mice: 8–12 weeks old. **DOI:** http://dx.doi.org/10.7554/eLife.12444.021

**Figure 8—figure supplement 3.**
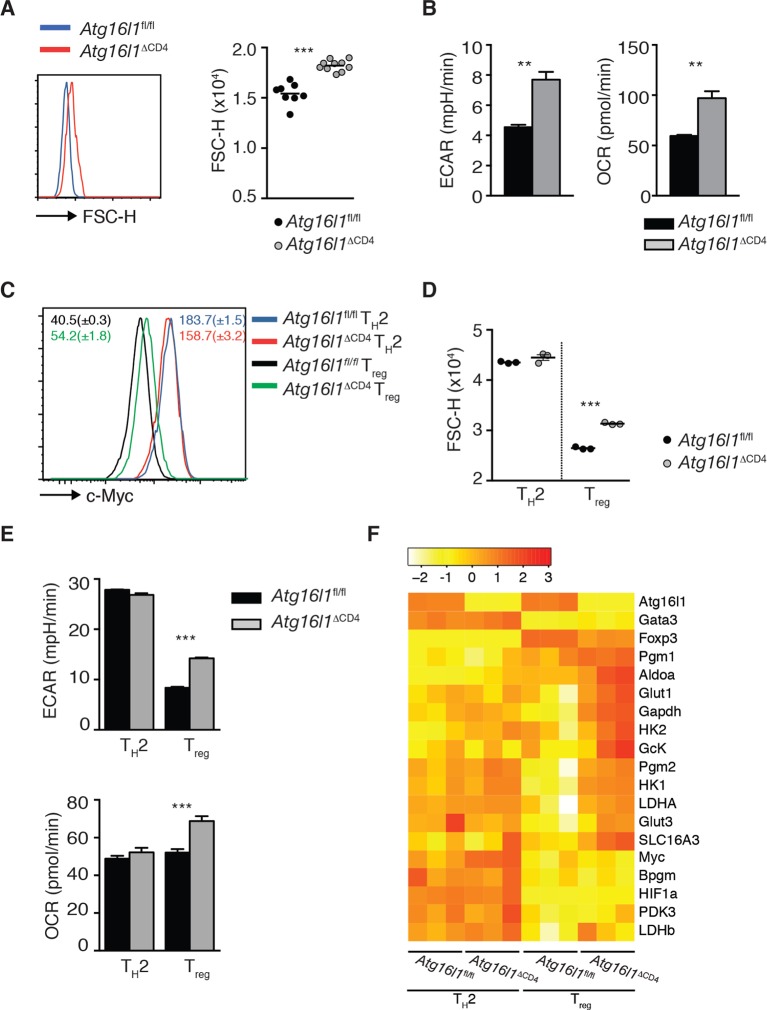
T_H_2 cells exhibit an enhanced glycolytic metabolic profile that is independent of autophagy. (**A**) Representative FACS plot and quantification of the cell size (FSC-H) of naïve (CD44^-^ CD62L^+^) CD4^+^ T cells from the spleen of *Atg16l1*^ΔCD4^ or *Atg16l1*^fl/fl^ littermates. (**B**) Basal level of oxygen consumption rate (OCR) and extracellular acidification rate (ECAR) in naïve (CD44^-^ CD62L^+^) unstimulated CD4^+^ T cells isolated from the spleen of *Atg16l1*^ΔCD4^ or *Atg16l1*^fl/fl^ littermates measured using the Seahorse metabolic flux analyzer. (**C,D**) Expression of c-Myc (**C**) and cell size (**D**) was analyzed by FACS in *Atg16l1*^ΔCD4^ or *Atg16l1*^fl/fl^ CD4^+^ T cells that were cultured in T_H_2 or T_reg-_polarizing conditions for 3 days and rested for one day in the presence of polarizing cytokines. (**E**) Basal level of OCR and ECAR were measured by Seahorse metabolic flux analyzer in *Atg16l1*^ΔCD4^ or *Atg16l1*^fl/fl^ CD4^+^ T cells cultured in T_H_2 or T_reg- _polarizing conditions for 3 days and rested for 2 days in the presence of polarizing cytokines. (**F**) qPCR analysis of glycolytic gene levels in *Atg16l1*^ΔCD4^ or *Atg16l1*^fl/fl^ CD4^+^ T cells cultured in T_H_2 or T_reg_ polarizing conditions for 3 days and rested for 1 day in the presence of polarizing cytokines. Data are combined from two independent experiments (**A**), or are representative of two independent experiments (**B-D**), or are from one experiment (**E,F**). Each dot represents an individual mouse (**A**) or individual cell culture (**D**). ECAR and OCAR data represent mean ± s.e.m values of T cell populations that were assayed in triplicates or quadruplicates (**B,E**). Gene expression data of triplicate cultures represent normalized expression values for each gene that were scaled to a mean of 0 and a standard deviation of 1 (**F**). Statistical significance was determined using the Mann–Whitney test (**A**) or unpaired Student’s t –test (**B,D,E**), **p<0.01; **p<0.001. **DOI:** http://dx.doi.org/10.7554/eLife.12444.022

### Differential survival of autophagy-deficient T_reg_ cells and T_H_2 cells is associated with an altered metabolic profile

Finally, we investigated mechanisms that might underlie the striking survival defect of *Atg16l1*-deficient intestinal T_reg_ cells. Analyses of key regulators of apoptosis revealed that *Atg16l1*-deficient T_reg_ cells isolated from spleen and cLP had comparable expression of pro-apoptotic (*Bim, Bax*) and anti-apoptotic (*Bcl2*) genes as those isolated from control mice (Figure 8—figure supplement 1B). As recent evidence suggests that tissue-resident T_reg_ cell populations may exhibit specialized metabolic adaptations (Burzyn et al., 2013), we compared the expression of metabolic genes by WT and *Atg16l1*-deficient T_reg_ cells. Analyses of genes involved in glycolysis, fatty acid synthesis (FAS) and fatty acid oxidation (FAO), revealed that *Atg16l1*-deficient T_reg_ cells had higher expression of glycolytic genes, including *Glut1, Slc16a3, Tpi1, Ldh-a, Aldo-a, Gpi1* and *Pgk1,* than control T_reg_ cells (Figure 8C). Strikingly, this augmented glycolytic signature was much more pronounced in *Atg16l1*-deficient T_reg_ cells isolated from cLP versus those from the spleen (Figure 8C). Conversely, expression of many key genes involved in FAS/FAO, including *Acc1, Acc2, Srebf1, Srebf2, Fdft1, Fabp1, Fabp4* and *Fabp5* was markedly decreased in *Atg16l1*-deficient T_reg_ cells (Figure 8D). Again, these differences were most pronounced in the intestine; WT cLP T_reg_ cells showed increased FAS/FAO gene expression compared to their spleen counterparts, whereas *Atg16l1*-deficient cLP T_reg_ cells were not able to up-regulate the expression of FAS/FAO genes (Figure 8D). Thus, *Atg16l1*-deficiency profoundly influenced the expression of metabolic genes in intestinal T_reg_ cells, with an altered balance of glycolytic and FAS/FAO gene expression. Further evidence of increased reliance on lipid metabolism by colonic T_reg_ cells was provided by our observation that T_reg_ cells isolated from the cLP showed markedly increased lipid uptake in comparison to mLN or spleen T_reg_ cells (Figure 8—figure supplement 2A,B). A similar pattern was observed when we assayed expression of CD36, a fatty acid translocase that enhances FA uptake: colonic T_reg_ cells showed increased expression of CD36 compared to splenic and mLN Treg cells (Figure 8—figure supplement 2C,D). Interestingly, we found that *Atg16l1*-deficient T_reg_ cells showed comparable levels of lipid uptake and CD36 expression as their autophagy-sufficient counterparts (Figure 8—figure supplement 2A–D), suggesting that autophagy does not affect lipid uptake per se but rather affects lipid metabolism.

Together, these results demonstrate that cell-intrinsic autophagy is indispensable for Foxp3^+^ T_reg_ cell maintenance and function in peripheral tissues, particularly to suppress inflammatory responses within the gastrointestinal tract. Decreased survival of *Atg16l1*-deficient T_reg_ cells was associated with an altered metabolic profile, suggesting that autophagy plays an integral role in facilitating the metabolic adaptions required for long-term T_reg_ cell survival in the intestine.

We next explored whether autophagy had a general effect on T cell metabolic profile and whether this might explain the differential effects on T_H_2 cells and T_reg_ cells. Evidence that this might be the case came from our observation that *Atg16l1*-deficient naïve CD4^+^ T cells exhibited increased cell size compared with naïve CD4^+^ T cells isolated from *Atg16l1*^fl/fl^ littermates (Figure 8—figure supplement 3A). We therefore measured oxygen consumption rate (OCR), which is an indicator of oxidative phosphorylation (OXPHOS), and extracellular acidification rate (ECAR), an indirect indicator of aerobic glycolysis. We found that *Atg16l1*-deficient naïve CD4^+^ T cells exhibited significantly increased OCR and ECAR, metabolic changes that are typically observed in activated CD4^+^ T cells and are associated with increased aerobic glycolysis (Figure 8—figure supplement 3B).

As T_H_2 cells have previously been reported to display an increased glycolytic rate compared to other T_H_ subsets (Michalek et al., 2011; Yang et al., 2013), we hypothesized that they may be more resistant to the increased glycolysis that is induced in the absence of autophagy. As it was not possible to sort T_H_2 cells from the cLP, we performed this analysis on in vitro cultures of T_H_2 and T_reg_ cells. We found that T_H_2 cells were larger than T_reg_ cells, expressed higher levels of c-Myc, a critical regulator of metabolic reprograming in activated T cells, and had markedly higher ECAR, all indicative of enhanced aerobic glycolysis (Figure 8—figure supplement 3C–E). Furthermore, while *Atg16l1*-deficient T_reg_ cells showed higher expression of c-Myc, significantly increased levels of ECAR and OCR, and were larger than their control *Atg16l1*-sufficient counterparts, we observed constitutively high and comparable levels of glycolysis in *Atg16l1*-deficient and *Atg16l1*-sufficient T_H_2 cells (Figure 8—figure supplement 3C–E). These patterns were recapitulated when expression of key metabolic genes were analyzed; T_H_2 cells showed high expression of a panel of glycolytic genes irrespective of their autophagy *Atg16l1* genotype, whereas T_reg_ cell expression of glycolytic genes was generally lower, unless the T_reg_ cells were autophagy-deficient (Figure 8—figure supplement 3F). Taken together, these results suggest that the enhanced glycolytic metabolism constitutively employed by T_H_2 cells makes them more resistant to the metabolic changes that occur in the absence of autophagy.

## Discussion

The unique challenges of the intestine necessitate complex mechanisms of tolerance and immune regulation to maintain homeostasis (Izcue et al., 2009). As altered mucosal CD4^+^ T cell responses are implicated in intestinal diseases of increasing prevalence, including food allergies and IBD (Maloy and Powrie, 2011; Berin and Sampson, 2013), it is important to understand the factors that control effector and regulatory T cell homeostasis in the gut. Here, we identify Atg16l1 and autophagy as a new critical pathway regulating intestinal T_reg_ and T_H_2 responses.

Recent studies addressing the role of autophagy in distinct leukocyte populations have highlighted T cells as being very sensitive to perturbations in the autophagy pathway (Ma et al., 2013). Our data extend these findings by showing that autophagy is particularly important for the survival of CD4^+^ T cells within the gut environment, as *Atg16l1* deletion in T cells led to a severe reduction of CD4^+^ T cell numbers in the intestinal LP. This deficit was confirmed in mixed bone marrow chimeras, where *Atg16l1*-deficient CD4^+^ T cells failed to reconstitute the intestinal LP compartment, and by the rapid outgrowth of adoptively transferred WT CD4^+^ T cells in the colonic LP of *Atg16l1*^ΔCD4^ recipients. However, despite the reduction in intestinal CD4^+^ T cells, *Atg16l1*^ΔCD4^ mice spontaneously developed progressive, chronic intestinal inflammation. To confirm their increased predisposition to develop intestinal pathology, we used an experimental model of IBD triggered by infection with *Helicobacter hepaticus* and concomitant treatment with anti-IL-10R mAbs (Song-Zhao and Maloy, 2014). This model induces severe typholocolitis that is T cell dependent and displays several features of human IBD pathology and does not require any specific genetic manipulation or chemical barrier disruption. We found increased intestinal pathology in *Atg16l1*^ΔCD4^ mice, confirming that *Atg16l1*-deficient T cells could mediate potent inflammatory responses in the gut. Thus, selective autophagy deficiency within T cells decreases the competitiveness of these cells and simultaneously predisposes to intestinal inflammation.

We found that *Atg16l1*^ΔCD4^ mice exhibited a drastic reduction in Foxp3^+^ T_reg_ populations in the cLP and SI LP, together with marked changes in intestinal T_reg_ phenotype, including increased cell cycling and aberrant production of T_H_ effector cytokines. The role of autophagy in Foxp3^+^ T_reg_ cells is not well defined. T cell-specific ablation of *Vps34,* which encodes a class III phosphatidylinositol 3-kinase that promotes autophagy, resulted in decreased frequencies of T_reg_ cells in the thymus, spleen and lymph nodes (Parekh et al., 2013). However, as Vps34 also has autophagy-independent functions (Backer, 2008), it was unclear as to what extent these changes were due to impaired autophagy. Furthermore, we did not find any deficit in thymic T_reg_ cell development in A*tg16l1*-deficient T cells. However, we observed that T_reg_ cells isolated from the mLN and colonic LP had increased levels of autophagy compared to effector T cells, suggesting that autophagy is particularly important for the maintenance of T_reg_ cells in the periphery. Indeed, we demonstrated that cell-intrinsic autophagy is indispensible for the maintenance and function of Foxp3^+^ T_reg_cells in the gastrointestinal tract, as selective deletion of *Atg16l1* in the Foxp3^+^ T_reg_ compartment in *Atg16l1*^ΔFoxp3^ mice led to a loss of intestinal Foxp3^+^ T_reg_ cells and to severe inflammation of the small intestine and colon. In this context, it is pertinent that rapamycin, which induces autophagy through its inhibitory activity on mTOR, has been shown to promote expansion of T_reg_ cells in vitro and in vivo (Pollizzi and Powell, 2015). Similarly, several small-molecule inducers of autophagy were shown to selectively promote the development of T_reg_ cells in vitro (Shaw et al., 2013). Taken together with our findings, these observations suggest that boosting autophagy may represent a rational therapeutic approach to enhance T_reg_ responses in the intestine.

How does autophagy intrinsically regulate T_reg_ cell homeostasis? Our data indicate that autophagy is not required for the differentiation of Foxp3^+^ T_reg_ cells in vitro or in vivo for thymic generation of T_reg_ cells in vivo. However, we found that *Atg16l1*-deficient T_reg_ cells showed significantly decreased survival compared to WT T_reg_ cells both in vitro and in vivo. As recent evidence indicates that T_reg_ cells utilize a distinct metabolic program that favors lipid oxidation for energy provision (MacIver et al., 2013), one potential explanation is that autophagy regulates T_reg_ cell metabolism and thereby their survival. Indeed, we found that *Atg16l1*-deficient T_reg_ cells expressed a distinct metabolic profile to their WT counterparts, exhibiting increased expression of genes involved in glycolysis and reduced expression of genes involved in FAS/FAO. Fatty acid metabolism is emerging as a potent regulator of T cell responses and preferential utilization of FAO has been linked to T_reg_ cell induction (Lochner et al., 2015). Although a recent report indicated that *de novo* FAS was not required for Foxp3^+^ T_reg_ cell differentiation (Berod et al., 2014), optimal in vivo T_reg_ cell function was associated with intrinsic lipid synthesis (Zeng et al., 2013). Furthermore, autophagy has been implicated in the regulation of fatty acid metabolism (Singh et al., 2009; Lizaso et al., 2013; Kaur and Debnath, 2015) and recent studies found that autophagy plays a key role in the generation of CD8^+^ memory T cells (Puleston et al., 2014; Xu et al., 2014), which are heavily dependent on FAO for survival (Pearce et al., 2009; O'Sullivan et al., 2014). Thus, autophagy could play a similar survival role in T_reg_ cells, by facilitating the degradation of intracellular lipid stores to release FAs that fuel FAO. Additionally, as degradation of intracellular lipids by autophagy is important to avoid lipotoxicity (Galluzzi et al., 2014), defective autophagy could lead to a toxic build up of intracellular lipids in intestinal T_reg_ cells.

The imbalance between glycolysis and FAS/FAO observed in autophagy-deficient T_reg_ cells could indicate that these cells have stalled in the activated/effector state and are unable to make the metabolic adaptations necessary for long-term survival. This is supported by our data showing that a higher proportion of autophagy-deficient T_reg_ cells appear to be in cell cycle, but they have reduced expression of terminal differentiation markers. Consistent with our findings, a recent study reported that autophagy deficiency in T_reg_ cells resulted in increased mTORC1 activation and glycolysis, leading to phenotypic instability, including expression of pro-inflammatory cytokines (Wei et al., 2016). However, the molecular mechanism behind decreased survival of autophagy-deficient T_reg_ cells was not elucidated (Wei et al., 2016). It is striking that autophagy deficiency had a more detrimental effect on intestinal T_reg_ cells than on those found in secondary lymphoid organs. Recent evidence suggests that tissue-resident T_reg_ cells undergo tissue-specific adaptations, and metabolic changes are emerging as an important facet of such reprogramming (Burzyn et al., 2013; Liston and Gray, 2014). Taken together, our results suggest that autophagy endows intestinal T_reg_ cells with the metabolic flexibility required to survive in the gut tissue, where essential growth factors may be in short supply (Pearce et al., 2013).

Paralleling decreased T_reg_ responses in *Atg16l1*^ΔCD4^ mice, we observed a selective expansion of T_H_2 cells in the intestinal LP that was already present in young mice and preceded the onset of overt pathology. Our subsequent analyses indicated that autophagy limits mucosal T_H_2 cells through both cell-intrinsic and cell-extrinsic (T_reg_-mediated) regulation. One possibility is that *Atg16l1*-deficient T_H_2 cells may be somewhat resistant to T_reg_ suppression. However, when we reconstituted pT_reg_ cells in *Atg16l1*^ΔCD4^ mice we observed a negative correlation between the numbers of intestinal T_reg_ cells and T_H_2 cells (data not shown), suggesting that autophagy-deficient T_H_2 cells are partially controlled by T_reg_ cells. Our data strongly suggest that the intrinsic survival advantage of *Atg16l*-deficient T_H_2 cells is primarily responsible for their outgrowth in the intestine. Indeed, we observed increased survival of *Atg16l1*-deficient T_H_2 cells in vitro, suggesting that autophagy might directly inhibit T_H_2 cell expansion. This concept is consistent with a previous study that reported enhanced survival of T_H_2 cells in vitro when autophagy was inhibited and that autophagy mediated death of T_H_2 cells during growth-factor withdrawal (Li et al., 2006). However, we provide evidence for an additional mechanism that could explain the preferential expansion of *Atg16l1*-deficient T_H_2 cells in the intestine, related to the unique ability of T_H_2 cells to cope with prolonged high levels of glycolysis. Our data contribute to accumulating evidence that a shift toward glycolysis is a general phenomenon observed when the autophagy pathway is perturbed in T cells. We observed characteristic signs of increased glycolysis in *Atg16l1*-deficient naïve CD4^+^ T cells and T_reg_ cells, such as increases in cell size, c-Myc levels and expression of glycolytic genes, as well as elevated ECAR. Others have reported a similar glycolytic shift in autophagy-deficient CD8^+^ memory T cells (Puleston et al., 2014) and T_reg_ cells (Wei et al., 2016). Interestingly, T_H_2 cells have previously been shown to display an increased glycolytic rate compared to other T_H_ subsets (Michalek et al., 2011; Yang et al., 2013). We confirmed the high levels of constitutive glycolysis in T_H_2 cells and showed that these were comparable in *Atg16l1*-deficient and control T_H_2 cells. Moreover, Gata3 activation was previously linked to induction of glycolysis after TCR activation in T cells, through induction of c-Myc, a critical regulator of metabolic reprograming (Wang et al., 2011; Wang et al., 2013; Wan, 2014). We therefore propose that in T_H_2 cells Gata3 orchestrates metabolic adaptations that enable these cells to cope with prolonged high levels of glycolysis, thus making them resistant to metabolic changes enforced by autophagy deficiency. Overall, our results indicate that autophagy is a key pathway through which T_H_2 responses are restrained in vivo. A lack of this restraint leads to a gradual loss of tolerance to intestinal antigens, as the excessive T_H_2 responses in *Atg16l1*^ΔCD4^ mice led to production of IgG_1_ and IgA antibodies toward commensal microbiota and dietary antigens that increased with age. Furthermore, *Atg16l1*^ΔCD4^ mice developed very high levels of circulating IgE, and mounted de novo IgE antibody responses toward introduced dietary antigen.

As polymorphisms in autophagy genes are linked to IBD susceptibility, our results point towards a novel mechanism that links impaired autophagy to intestinal inflammation through dysregulation of mucosal T cell responses. Previous studies focused on the role of *ATG16L1* and autophagy in myeloid cells and the intestinal epithelium. They suggested that impaired autophagy could result in reduced intestinal barrier integrity due to impaired Paneth cell function within the intestinal epithelial layer and elevated cytokine responses by macrophages and dendritic cells (Cadwell et al., 2008; Saitoh et al., 2008; Lassen et al., 2014). Our data add a further layer to the control of intestinal homeostasis by autophagy, by showing that autophagy impairment alters the local T cell compartment and promotes T cell driven intestinal pathology. We present compelling evidence that autophagy deficiency in T_reg_ cells leads to a deficit in intestinal T_reg_ cells and the development of severe intestinal pathology. Although the contribution of the T_H_2 axis to IBD remains unclear (Strober et al., 2002; Shale et al., 2013), polymorphisms in *IL-4, IL-5* and *IL-13* have been implicated by GWAS in both CD and UC (Van Limbergen et al., 2014) and elevated levels of antibodies recognizing food and commensal antigens have been detected in IBD patients (Lodes et al., 2004; Cai et al., 2014). Moreover, as defective T_reg_ and increased T_H_2 responses at the mucosa are observed in food allergies and asthma, our findings might also have implications for these conditions. Indeed, epidemiological studies show an overlap between IBD and T_H_2 driven diseases, such as atopic dermatitis and asthma (Lees et al., 2011). Furthermore, polymorphisms in the essential autophagy gene *Atg5* have recently been implicated in asthma susceptibility (Martin et al., 2012; Poon et al., 2012). Autophagy is an attractive therapeutic target and several autophagy modulating compounds are already in clinical trials for the treatment of various disorders (Jiang and Mizushima, 2014). Furthermore, natural dietary-derived compounds, including retinoid acid (Isakson et al., 2010) and vitamin D (Yuk et al., 2009), have been shown to enhance autophagy. Taken together with our results, these findings raise the possibility that activation of autophagy through dietary or pharmacological modulation might have beneficial effects in disorders with a signature of decreased T_reg_ and elevated T_H_2 responses, including intestinal inflammation and various hypersensitivities.

## Materials and methods

### Mice

*Atg16l1*^fl/fl^ mice were generated and provided by the H. Virgin laboratory (Washington University, Saint Louis, MO), as described (Hwang et al., 2012). *Atg16l1*^fl/fl^ mice were crossed to B6.Cg-Tg(Cd4-cre)1Cwi/BfluJ (*CD4-Cre* mice) and B6.129(Cg)-Foxp3^tm4(YFP/cre)Ayr^/J (*Foxp3*^Cre^ mice, Jackson Laboratory, Bar Harbor, ME) to generate *Atg16l1*^ΔCD4^ and *Atg16l1*^ΔFoxp3^ mice, respectively. All above strains, together with B6.SJL-CD45.1 (CD45.1^+^), B6 *Rag1*^-/-^ (Jackson Laboratory), and B6 *Foxp3*^hCD2^ mice (Komatsu et al., 2009) were bred and maintained under specific pathogen-free conditions. Unless stated otherwise, mice were analyzed at 8–12 weeks (young mice) or > 5 months of age (aged mice). In the gene expression analysis *Atg16l1*^ΔFoxp3^ mice and *Foxp3*^Cre^ mice were co-housed and age- and sex- matched. In all other experiments mice used were age- and sex-matched littermates that were kept co-housed throughout the experiments.

### T cell-mediated colitis

Experimental T cell-mediated colitis was induced by infection with *Helicobacter hepaticus* and concomitant IL-10R blockade as described (Song-Zhao and Maloy, 2014). Briefly, mice were infected with *H.hepaticus* (10^8^ CFU per mouse) by oral gavage on three consecutive days and anti-IL-10R mAb (1B1.2) was administrated via i.p. injection (1 mg per mouse) on the first and seventh day of the infection. Mice were sacrificed 2 weeks after colitis induction.

### Histological assessment of intestinal inflammation

Mice were euthanized at indicated time points whereupon tissue sections were fixed in buffered 10% formalin and paraffin-embedded. Sections were then cut and stained with hematoxylin and eosin. Histological analysis of intestinal inflammation was performed as described (Song-Zhao and Maloy, 2014). Briefly, inflammation was graded semi-quantitatively on a scale from 0 to 3, for four criteria; (a) epithelial hyperplasia and goblet cell depletion, (b) lamina propria leukocyte infiltration, (c) area of tissue affected, and (d) markers of severe inflammation, including crypt abscesses, sub- mucosal inflammation, and ulceration. Scores for individual criteria were totaled for an overall inflammation score between 0 and 12.

### Isolation of cells and flow cytometry analysis

Cell suspensions were prepared from the thymus, spleen, mLN, bone marrow and intestinal lamina propria as previously described (Uhlig et al., 2006). The following antibodies from eBioscience (Hatfield, UK) were used: anti-CD16/32 (93), anti-CD4 (GK1.5), anti-CD8α (53.6.7), anti-TCRβ (H57-597), anti-CD45 (30-F11), anti-CD44 (1M7), anti-CD62L (MEL-14), anti-CD45.1 (A20), anti-CD45.2 (104), anti-CD103 (2E7)), anti-CD69 (H1.2F3), anti-KLRG1 (2F1), anti-CD25 (7D4), anti-CD36 (No.72–1), anti-hCD2 (RPA-2.10), anti-CTLA4 (UC10-4B9), anti-GR.1 (RB6-8C5), anti-CD11b (M1/70), anti-Siglec F (E50-2440), anti-Gata3 (TWAJ), anti-Foxp3 (FJK-16s), anti-Ki67 (SolA15), anti-Helios (22F6), anti- Bcl2 (10C4), anti-PS6 (cupk43k), anti-IFN-γ (XMG1.2), anti-IL-17A (eBio17B7), anti-IL-13 (eBio13A). The following antibodies were from BioLegend (San Diego, CA): anti-CD138 (281–2), anti-CD161 (PK136), anti-F4/80 (BMB), anti-CD11b (M1/70). The following antibodies were from BD Biosciences (San Jose, CA): anti-B220 (RA3 6B2), anti-GL7 (GL7), anti-CD95 (Jo2), anti-CD3 (145-2C11), anti-CD19 (1D3), anti-Ly6C (AL-21), anti-Ly6G (1A8), anti-IgM (R6-60.2), anti-IgG1 (A85-1). Anti-c-Myc antibody was from Cell Signaling Technology (D84C12, Danvers, MA). Anti-Neuropilin1 polyclonal antibody was from R&D Systems (FAB566A, Minneapolis, MN). Fixable Viability Dye from eBioscience was used to stain dead cells. Annexin V staining was performed using eBioscience kit (88–08006) according to manufacture instructions. For intracellular cytokine staining cells were stimulated for 3h with PMA (100ng/ml) and Ionomycin (1 µg/ml) in the presence of Brefeldin A (10 µg/ml).

Autophagosome formation detection by flow cytometry was performed using FlowCellect Autophagy LC3 Antibody-based Assay Kit (FCCH100171, Merk-Millipore, Billerica, MA) according to the manufacturer's instructions and following cell surface markers staining. The Autophagy LC3 Antibody-based Assay Kit involves a permeabilization step to wash out cytosolic LC3-I, allowing for antibody-based detection of membrane bound LC3-II. For autophagy detection in WT T_reg_ cells B6 Foxp3^hCD2^ were used, as this allowed the detection of Foxp3^+^ T_reg_ cells on the basis of surface expression of hCD2 marker. All data were acquired using a Cyan ADP (Beckman Coulter, High Wycombe, UK) and analyzed using FlowJo software (Tree Star, Ashland, OR).

### CD4^+^ T cell purification

Bulk CD4^+^ T cells were purified from the spleen and mLN by negative selection as previously described (Coccia et al., 2012). Naïve CD4^+^ T cells were then sorted as CD4^+^ CD25^-^ CD44^-^ CD62L^+^. T_reg_ cells were sorted as CD4^+^ CD25^+^ when sorted from *Atg16l1*^ΔCD4^ and *Atg16l1*^fl/fl^ mice and as CD4^+^ YFP^+^ when sorted from *Atg16l1*^ΔFoxp3^ and *Foxp3*^Cre^ mice. Cells were sorted using an Astrios, Beckman Coulter MoFlo XDP or AriaIII BD Bioscience. Post-sort flow cytometry analyses confirmed that the purity of sorted populations was >97%.

### Adoptive transfer of naïve CD4^+^ T cells

Naïve CD4^+^ T cells from WT (CD45.1^+^) mice were sorted as described above and transferred to *Atg16l1*^ΔCD4^ recipient (CD45.2^+^) mice via intravenous injection (4-5x10^6^ cells per mouse). Analysis of spleen, mLN and cLP CD4^+^ T and T_reg_ cells was performed 3 months after transfer.

### Generation of mixed bone marrow chimeras

BM cells were isolated from the tibia and femur of WT (CD45.1^+^) mice and *Atg16l1*^fl/fl^ or *Atg16l1*^ΔCD4^ (CD45.2^+^) mice and injected i.v. at 1:1 ratio (a total of 1x10^7^ cells per mouse) into lethally irradiated (1100 Rad, split dose) *Rag1*^-/-^ recipients. Mice were allowed to reconstitute for at least 8 weeks before analysis.

### Immunization with ovalbumin (OVA)

For induction of OVA-specific IgE antibodies two treatment regimes were utilized. For OVA only immunization mice were fed three times by oral gavage with ovalbumin grade VII (5 mg per mouse, Sigma-Aldrich, St Louis, MO) with 21-day intervals between feeds. For adjuvanted immunization, mice were initially fed with OVA (5 mg per mouse) plus cholera toxin (10 μg per mouse, Biologial Compbell), after which they were fed twice with OVA only (5mg per mouse), with 21-day intervals between feeds.

### Infection with *Trichuris muris* and detection of *T. muris-*specific IgG_1_

Mice were orally infected with ~200 *Trichuris muris* eggs. Serum was collected on day 34-post infection and assayed by ELISA for parasite-specific IgG_1_. Ninety-six-well plates were coated with 5 μg/ml *T. muris* excretory/secretory antigen and incubated with serial two-fold diluted serum. Bound IgG_1_ was detected using biotinylated anti-murine IgG_1_ (AbD Serotec, Kidlington, UK).

### Lipid uptake measurement

*Atg16l1*^fl/fl^ and *Atg16l1*^ΔCD4^ mice were injected i.p. with 50 μg of fluorescent 16-carbon fatty acid analog BODIPY C-16 (Molecular Probes) reconstituted in DMSO. Mice were culled 1 hr later and tissue collected for analysis by flow cytometry.

### Metabolic analysis using XF 96 extracellular flux analyzer

The real-time extracellular acidification rate (ECAR) and oxygen consumption rate (OCR) were measured using a XF 96 extracellular flux analyzer (Seahorse Bioscience, Billerica, MA). Briefly, naïve (CD62L^+^CD44^-^) CD4^+^ T cells, or in vitro polarized T_H_2 and T_reg_ cells, were washed twice in assay medium (RPMI 1640 without sodium bicarbonate, 20 mM glucose, 1% FCS, 2mM pyruvate) and seeded at 3–4 x 10^5^ cells per well in assay medium in a 96-well XF plate coated with poly-L-lysine (Sigma). T cells were rested for 1 hr at 37°C without CO_2_ before analysis.

### Polarization and stimulation of CD4^+^ T cell subsets

Naïve CD4^+^ T cells were cultured (3x10^5^ cells/well) in 96-well plates coated with anti-CD3 mAb (5 μg/ml) and soluble anti-CD28 mAb (1 μg/ml) and kept in presence of IL-2 (100 U/ml). For T_H_0 conditions anti-IL-4 (10 μg/ml) and anti-IFN-γ (10 μg/ml) mAb were added. Cultures were supplemented with IL-12 (10 ng/ml) and anti-IL-4 mAb (10 μg/ml) for T_H_1 polarization; with IL-4 (20 ng/ml), anti-IFN-γ (20 μg/ml) and anti-IL-12 (10 μg/ml) for T_H_2 polarization; and with TGF-β1 (5 ng/ml), anti-IFN-γ, anti-IL-4 mAb and anti-IL-12 (all 10 μg/ml) for induced T_reg_ polarization. Sorted T_reg_ cells were activated for 48h with anti-CD3 mAb (5 μg/ml) and soluble anti-CD28 mAb (1 μg/ml) plus IL-2 (100 U/ml) and then cultured with IL-4 (10 ng/ml), IL-13 (10 ng/ml) and IL-2 (100 U/ml) for 5 days. All cytokines were from R&D Systems. Anti-CD3 (145-2C11), anti-CD28 (37.51), anti-IFN-γ (XMG1.2), anti-IL-12 (C17.8) and anti-IL-4 (11B11) mAb were from eBioscience. Cells were cultured in RPMI-1640 Medium, 10% fetal calf serum, 2 mM L-glutamine, 100 U/ml of Penicillin/Streptomycin, and 0.05 mM 2-mercaptoethanol.

### Measurement of serum antibodies and cytokines

All immunoglobulin isotypes except for IgE were measured by enzyme-linked immunosorbent assay (ELISA) using the SBA Clonotyping System (Southern Biotech, Birmingham, AL). IgE concentration was determined using an anti-mouse IgE ELISA (BioLegend), according to manufacturer's instructions. For the detection of soy-specific, CBir-specific and *Helicobacter*-specific antibodies ELISA was performed with plates coated with purified soy antigen (5 μg/ml), CBir peptide (10 μg/ml) and soluble *Helicobacter* antigen (sHel antigen, 10 μg/ml) respectively. sHel antigen was prepared as previously described (Kullberg et al., 1998). For the detection of OVA-specific IgE, a sandwich ELISA was performed with biotinylated-OVA used for detection. MCPT-1 concentrations were measured by ELISA (eBioscience).

### Immunofluorescence microscopy

Colonic and small intestine tissue samples were formalin-fixed, paraffin-embedded and sectioned as per histological analysis. Sections were deparaffinized, rehydrated, and subjected to sodium citrate-based antigen retrieval, then stained with mouse pAb anti-β-catenin (610153, BD Bioscience), rabbit pAb anti-CD3 (ab5690, Abcam, Cambridge, UK) and secondary goat antibodies conjugated to AlexaFluor488 or 555 (Life Technologies, Carlsbad, CA). Slides were mounted with DAPI-containing Vectashield (Vector Laboratories, Burlingame, CA). Images were acquired with an Olympus Fluoview FV1000 confocal microscope and Olympus Fluoview Software (Olympus, Tokyo, Japan).

### Western blotting analysis

CD4^+^ T cells purified by negative selection were lysed in RIPA buffer containing protease inhibitor cocktail (Roche, Basel, Switzerland). Protein levels were normalized by Biorad DC protein assay (Bio-Rad Laboratories, Hercules, CA), resolved by SDS-PAGE and, following transfer onto nitrocellulose membranes, were blotted with anti-LC3 antibody (L7543; Sigma-Aldrich) and anti-tubulin antibody (sc5286, Santa Cruz Biotechnology, Dallas, TX), and secondary HRP conjugated anti–rabbit antibody (7074S, Cell Signaling Technology).

### Fluidigm gene expression analysis

CD4^+^ T cells and T_reg_ cells were sorted for each population based on surface marker and YFP expression from spleen and cLP of *Atg16l1*^ΔFoxp3^ and *Foxp3*^Cre^ mice. Two hundred cells/population were sorted in triplicates from a total of four (spleen) or six (cLP) mice per group. Alternatively, 250 cells from in vitro polarized populations of T_H_2 and T_reg_ cells were sorted from triplicate culture wells. RNA was reverse transcribed and cDNA was pre-amplified using the CellsDirect OneStep q-RT kit (Invitrogen). The selected autophagy, apoptotic and metabolic genes were amplified and analyzed for expression using a dynamic 48x48 array (Biomark Fluidigm) as previously described (Tehranchi et al., 2010). Data were analyzed using the 2^-∆Ct^ method, and the results were normalized to actin or HPRTprt expression.

### Statistical analysis

For weight curves and antibody titers, p-values were determined by two-way ANOVA with Bonferroni post-tests. For the metabolic analysis using XF 96 extracellular flux analyzer, p-values were determined using unpaired Student’s t-test. For all other experiments, p-values were determined by nonparametric Mann–Whitney test. Differences were considered statistically significant when p<0.05 (*<p0.05, **p<0.01, ***p<0.001). Data are shown as mean ± s.e.m. Statistics were calculated using GraphPad Prism 6 software. For in vivo experiments, sample size was determined by power analysis using power of trial software, which calculates a power value based on X^2^ test statistics. Calculated required sample sizes were applied whenever possible. No mouse was excluded from the analysis. With the exception of histological assessment of intestinal inflammation, experimenters were not 'blinded' to allocation of animals to experimental groups.
